# Psychosocial interventions for anxiety disorders in adults: evidence mapping and guideline appraisal

**DOI:** 10.3389/fpsyt.2025.1677705

**Published:** 2025-10-20

**Authors:** Chuxian Huang, Xiao Liu, Yue Liu, Chen Luo, Jiajia Cai, Yanmei Wang, Jue Chen, Zhongying Shi, Xiaochao Jin

**Affiliations:** ^1^ Clinical Psychology Department, Nursing Department, Shanghai Mental Health Center, Shanghai Jiao Tong University School of Medicine, Shanghai, China; ^2^ Social Work Department, Shanghai Municipal Hospital of Traditional Chinese Medicine, Shanghai University of Traditional Chinese Medicine, Shanghai, China; ^3^ General Surgery,Nursing Department, Xinhua Hospital, Shanghai Jiao Tong University School of Medicine, Shanghai, China; ^4^ General Psychiatry Department, Nursing Department, Shanghai Mental Health Center, Shanghai Jiao Tong University School of Medicine, Shanghai, China

**Keywords:** anxiety disorder, guidelines, psychosocial intervention, systematic review, recommendations

## Abstract

**Introduction:**

This systematic review summarizes the recommendations related to psychosocial interventions for anxiety disorders included in existing guidelines and compares their differences.

**Methods:**

Computer-based searches were conducted to identify relevant guidelines on psychosocial interventions for anxiety disorders from domestic and international guideline websites, professional association websites, and other relevant databases. The guidelines’ quality was evaluated using the Appraisal of Guidelines for Research and Evaluation II (AGREE II) tool.

**Results:**

Fourteen guidelines from nine countries were included, with AGREE II scores ranging between 64.4%–96.3%. The specific recommendations were synthesized into a single evidence map, revealing that cognitive behavioral therapy demonstrated strong support for treating generalized anxiety, panic, and social anxiety disorders. Conversely, eye movement desensitization and reprocessing, exposure therapy, and virtual reality exposure therapy were not recommended for panic disorder. Additionally, no guidelines provided any recommendations for psychosocial interventions for separation anxiety disorder.

**Discussion:**

Guidelines on psychosocial interventions for adult anxiety disorders vary remarkably concerning their quality and recommended suggestions. Future guideline development or updates should strictly adhere to standardized development processes. Additionally, researchers should double their efforts to continuously explore and validate the efficacy of various psychosocial interventions in anxiety populations.

**Sytematic review registration:**

https://www.crd.york.ac.uk/PROSPERO/view/CRD420250654358, PROSPERO, identifier CRD420250654358.

## 1 Introduction

Anxiety disorders predominantly consist of generalized anxiety disorder, panic disorder, agoraphobia, specific phobia, social anxiety disorder, separation anxiety disorder, and selective mutism. These conditions are characterized by excessive fear, worry, and associated behavioral disturbances (1). Over the past three decades, China has undergone unprecedented economic development and social transformation. This profound shift has exposed the population to a range of challenges spanning work, education, cultural perceptions, and social norms. Notably, this societal context may be linked to the rising prevalence of anxiety disorders. Currently, anxiety disorders rank as the mental illness with the highest lifetime prevalence rate in China, at 7.57% (2). Globally, anxiety disorders also remain highly prevalent. The World Health Organization report demonstrates that approximately 301 million people suffer from anxiety disorders (3). Moreover, anxiety disorders frequently present alongside other mental or physical health conditions. Among psychiatric comorbidities, major depressive disorder is the most prevalent, as noted by Penninx, Pine, Holmes and Reif (1), and can increase the severity of anxiety disorders to a certain extent. Given that individuals with anxiety disorders typically experience persistent fear and worry, the condition is often associated with impairments across critical domains such as personal life, family functioning, social participation, and career development. Without timely intervention, it may further lead to functional deficits in areas including academic performance, cognitive functioning, decision-making capabilities, and attention span—ultimately impairing these individuals’ ability to carry out daily activities.

Presently, the treatment modalities for anxiety disorders largely include pharmaceutical treatment, psychosocial interventions, physical rehabilitation therapies, and management of comorbid diseases. Among these, selective serotonin reuptake inhibitors are regarded as the first-line recommendation for clinical treatment (4). However, long-term use of selective serotonin reuptake inhibitors can lead to adverse reactions, such as lethargy and decreased appetite; studies have confirmed that more than 50% of patients with anxiety are non-responsive to pharmacological treatment (5). Therefore, psychosocial interventions—classified as first-line treatment—have assumed an increasingly critical role in anxiety management. Certain guidelines (6, 7) indicate that psychosocial interventions can replace or partially substitute pharmacological therapy, with their primary objectives being to alleviate core symptoms, such as anxiety, tension, and fear; thereby, improving patients’ quality of life. Compared to routine care, various psychosocial interventions, such as cognitive behavioral therapy (CBT; Papola et al. (8)) and psychodynamic therapy (9), can enhance patients’ coping skills and psychosocial functioning while preventing relapses. It is important to note that psychosocial interventions do not share identical goals. For instance, CBT typically centers on symptom reduction, whereas acceptance- and mindfulness-based interventions—such as Acceptance and Commitment Therapy (ACT)—place greater emphasis on enhancing psychological flexibility and values-based living, rather than directly eliminating symptoms. Additionally, research has examined differences in efficacy among various psychosocial interventions for mixed anxiety disorders. For example, one randomized controlled trial (10), randomly assigned 128 participants diagnosed with at least one anxiety disorder subtype (e.g., generalized anxiety disorder, social anxiety disorder) to either the ACT group or the CBT group. Results revealed that participants in both groups exhibited similar overall reductions in anxiety levels before and after treatment. This study suggests that goal-oriented psychosocial interventions with different foci may all exert positive effects on individuals with mixed anxiety disorders, and their beneficial impacts are not limited to a specific type of anxiety disorder.

To date, clinical research has not reported any serious side effects of psychosocial interventions. Their gentle and sustainable characteristics provide universally applicable and safe treatment options for patients with diverse pathological features. In addition to alleviating anxiety symptoms across cognitive, emotional, and social functional domains, these interventions support long-term rehabilitation and improve both clinical efficacy and quality of life. Therefore, psychosocial interventions should be prioritized as core approaches by clinical practitioners in the treatment protocols for anxiety disorders.

Numerous authoritative organizations worldwide have published clinical practice guidelines for anxiety disorder treatment, which include several psychosocial interventions to assist healthcare providers and patients in making appropriate healthcare decisions in specific clinical contexts. However, these guidelines differ significantly across countries and organizations, and may even offer conflicting recommendations (11). This undermines the value of the guidelines for clinical practice and reduces the compliance of healthcare providers and patients with the recommended interventions. Furthermore, the quality of clinical guidelines is highly contingent on the rigor of their development process. Should deficiencies exist in this process—such as in evidence synthesis, consensus development, or conflict of interest management—it may directly undermine the guidelines’ reliability and applicability. Conducting a systematic review of guidelines within this field is therefore essential. Currently, the Appraisal of Guidelines for Research & Evaluation II (AGREE-II) (12) is widely recognized and adopted as the gold standard for guideline quality assessment in the international evidence-based medicine community. This tool delivers an objective, reproducible, and standardized evaluation of guideline quality across six core domains: Scope and Purpose, Stakeholder Involvement, Rigor of Development, Clarity of Presentation, Applicability, and Editorial Independence—thereby providing a scientific basis for determining guideline quality.

In summary, this study aims to collate international guidelines on anxiety disorders and conduct a systematic review to examine whether the existing guidelines include content on psychosocial interventions, while comparing the methodological quality and recommendations of specific psychosocial interventions mentioned in these guidelines. The goal is to provide a basis and reference for the development of subsequent relevant guidelines and practical decision-making to improve the clinical outcomes for patients with anxiety disorders.

## 2 Materials and methods

### 2.1 Study design and registration

This systematic review followed the updated Preferred Reporting Items for Systematic Reviews and Meta-Analyses (PRISMA 2020) statement and was recorded on PROSPERO (CRD420250654358), with our protocol being released later (13).

### 2.2 Literature search strategy

In this investigation, we systematically scoured domestic and international guideline websites, professional association websites, and relevant databases. Domestic and international guideline websites included UpToDate, BMJ Best Practice, Guidelines International Network, National Institute for Health and Care Excellence, Scottish Intercollegiate Guidelines Network, New Zealand Guidelines Group, Agency for Healthcare Research and Quality, Registered Nurses’ Association of Ontario, and Medsci Guidelines.

Professional association websites included the American Psychiatric Association, Anxiety and Depression Association of America, United States Preventive Services Task Force, Royal Australian and New Zealand College of Psychiatrists (RANZCP), NSW Ministry of Health, Australian Department of Health and Aged Care, World Federation of Societies of Biological Psychiatry (WFSBP), Chinese Medical Association, and Chinese Association of Integrative Medicine.

Databases included PubMed, Web of Science, Embase, CINAHL, Cochrane Library, Joanna Briggs Institute Database, Wanfang Data Knowledge Service Platform, China National Knowledge Infrastructure, Chinese Biomedical Literature Service System, and Chongqing VIP database. Both MeSH and free-text terms were combined and adopted. English search keywords included “anxiety disorders/anxiety” and “guideline*/consensus*/recommendation*.” Chinese search keywords included “anxiety/anxiety disorders” and “guideline*/consensus*/recommendation*.” The extent of the search went from the creation of the database to January 2025.

### 2.3 Inclusion and exclusion criteria

Guideline screening was independently conducted by two researchers (Huang and Jin). Inclusion criteria comprised articles published in Chinese or English that addressed psychosocial interventions for anxiety disorders. Exclusion criteria comprised duplicate records or superseded guidelines and guideline interpretations, translations, or post-implementation evaluations. Different reports from the same study can be found in the **Supplementary Material** (List of Different Reports from the Same Study).

### 2.4 Literature screening and data extraction

Two researchers, trained in the standardized evidence-based approach, systematically hunted for relevant literature. Studies were logged into the NoteExpress software to eliminate duplicates. Using the Population & Clinical Areas, Interventions, Comparators, Attributes of CPGs, and Recommendation characteristics (PICAR) (14, 15) framework (**Table 1**), two researchers independently screened titles and abstracts based on the inclusion and exclusion criteria. Finally, the full text was read for rescreening. Data were extracted using a specially designed basic information extraction table and a content extraction table, and then cross checked. The basic information extraction form for the guidelines included authors, guideline title, type, country/region, target population, and development/update date. The recommendation content extraction form included types of anxiety disorders addressed in the guideline, recommended specific psychosocial interventions, and recommendation strength.

**Table 1:** Eligibility criteria pertaining to the population & clinical areas, interventions, comparators, attributes of CPGs, and recommendation characteristics (PICAR) framework.

PICAR element	Study specific criteria
Population & Clinical area(s)	-Adult (>18 years) with anxiety disorder -Seven clinical indications: Treatment of: 1) generalized anxiety disorder; 2) panic disorder; 3) agoraphobia; 4) specific phobia; 5) social anxiety disorder; 6) separation anxiety disorder; 7) selective mutism
Interventions	-A series of social and psychosocial intervention measures such as CBT, IPT, ACT
Comparators	-No comparator
Attributes of guidelines	- Language: English and Chinese language - Publishing region: Global scope, no country specified - Version: Only the latest version of guidelines is of interest - Development process: Guidelines are explicitly evidence-based - System of rating evidence: Guidelines use a system to rate the level of evidence behind recommendations - Scope: Guidelines primarily focus on psychosocial interventions in adults with anxiety disorder - Recommendations: Guidelines will only be included if they report one or more eligible recommendations of interest
Recommendation characteristics	- Interventions: Recommendations must explicitly discuss at least one psychosocial intervention - Comparator(s): Recommendations are not required to compare an intervention of interest to another psychosocial intervention - Levels of confidence: Each recommendation must be accompanied by an explicit level of confidence (e.g., GRADE 1A)

### 2.5 Quality evaluation of included guidelines

In this study, we utilized the Appraisal of Guidelines for Research and Evaluation II (AGREE II (12);) to examine and determine the methodological quality of the guidelines that had been included. Four researchers (Huang, Jin, Luo, and Cai) independently conducted the evaluations after receiving training on AGREE II scoring criteria. Each included guideline was rated item-by-item according to the AGREE II criteria, with researchers documenting their rationales for each score. To determine the inter-rater reliability, the intraclass correlation coefficient (ICC (28);) was utilized. The descriptions for the ICC values were presented in the following manner: ICC < 0.40 indicated low consistency, 0.40 ≤ ICC < 0.75 indicated average consistency, and ICC ≥ 0.75 indicated high consistency.

AGREE II comprises 23 articles that are divided into 6 domains, consisting of “Scope and Purpose,” “Stakeholder Involvement,” “Rigor of Development,” “Clarity of Presentation,” “Applicability,” and “Editorial Independence.” Every item is evaluated using a scale ranging from 1 to 7. A rating of 1 signifies total non-conformance, whereas a rating of 7 represents full conformance. The standardized scores for each area are identified as the ratio of the highest possible score for that area expressed as a percentage. The calculation for the standardized score is [(actual score – minimum possible score)/(maximum possible score – minimum possible score)] x 100%. A higher score implies a higher standard of the guideline in that area. According to the standardized scores of each field in the guidelines, the recommendations are divided into three levels: Recommended: Six areas with scores ≥60% are rated as level A, Recommended with modifications: There are ≥3 fields with a score of ≥30%; however, <60% of the fields are rated as level B, and Not recommended: Fields with scores <30% and ≥3 are rated as level C.

### 2.6 Integration of the recommendations in the guidelines

Two researchers integrated the recommended situations into the guidelines extracted from the content extraction form of the guidelines’ recommendations. Among them, the recommended situations were classified into “strong recommendation,” “recommendation,” “unclear recommendation,” “non-recommendation,” and “not mentioned.” Additionally, we constructed a bubble chart to display the recommended situations of psychosocial interventions for various kinds of anxiety disorders stated in each guideline. Different types of anxiety disorders were presented on the Y-axis, and different types of psychosocial interventions were presented on the X-axis. Four colored spheres, namely green (strong recommendation), blue (recommendation), red (non-recommendation), and yellow (unclear recommendation), were used to distinguish between and visualize the recommended situations of the psychosocial interventions for different types of anxiety disorders.

## 3 Results

### 3.1 Study selection

The database investigation initially retrieved 2,087 works, which were reduced to 117 after the elimination of duplicates and the exclusion of irrelevant records by title and abstract. A full-text assessment was conducted for these 117 works. Following the application of the inclusion and exclusion criteria (**Supplementary Figure 1**), numerous articles were excluded. Finally, 14 guidelines from 12 organizations were incorporated into this research (6, 7, 16–27).

### 3.2 Characteristics of the guidelines

The characteristics of the incorporated guidelines are presented in **Table 2**. The publication years of these guidelines spanned from 2003 to 2023 in China (n = 3), Argentina (n = 1), Australia and New Zealand (n = 2), the UK (n = 2), Singapore (n = 1), Canada (n = 2), Germany (n = 1), Japan (n = 1), and India (n = 1); 35.7% of these guidelines were updated versions. None of the 14 guidelines included all types of anxiety. Of these, generalized anxiety disorder was included in 10 guidelines, social anxiety disorder in 9, specific phobia in 5, panic disorder in 6, panic disorder with agoraphobia in 4, SM in one, and separation anxiety disorder in two. Four guidelines used the Grading of Recommendations Assessment, Development, and Evaluation system, whereas four guidelines did not report on the strength of the recommendations. The funding information was reported in eight guidelines.

**Table 2:** Characteristics of the clinical practice guidelines.

Author	Year	Guideline theme	Version	Country/ Region	Primary developer/Publishing entity	Types of anxiety disorders (Diagnosis)	Strength of the recommendations	Guideline page	Funding
Shi et al. (7).	2023	Guidelines for the Prevention and Treatment of Anxiety Disorders in China (Second Edition)	Updated	China	CSP–CMA	GAD, PD, SAD (ICD-11 & DSM-V)	Not reported	416	None
Bandelow et al. (6)	2023	World Federation of Societies of Biological Psychiatry guidelines for the treatment of anxiety, obsessive-compulsive, and posttraumatic stress disorders-Version 3. Part I: Anxiety disorders	Updated	Argentina	WFSBP	PDA, GAD, SAD, SP, SM (ICD-10/ICD-11 & DSM-V)	The WFSBP evidence grading system	39	None
Andrews et al. (16)	2018	Royal Australian and New Zealand College of Psychiatrists clinical practice guidelines for the treatment of panic disorder, social anxiety disorder, and generalized anxiety disorder	Original	Australia & New Zealand	RANZCP	PDA, SAD, GAD (DSM-V)	EBR & CBR	64	Funding from RANZCP
NICE (17)	2020	Generalized anxiety disorder and panic disorder in adults: Management	Updated	The United Kingdom	NICE	GAD, PD (DSM-IV & DSM-IV-TR)	GRADE	47	Funding from NICE
MOH (18)	2015	Ministry of Health Clinical Practice Guidelines: Anxiety disorders	Updated	Singapore	MOH	PD, GAD, SP, SAD (ICD-10 & DSM-IV-TR)	GRADE	100	None
CPA (19)	2006	Management of anxiety disorders	Original	Canada	CPA	PD, SAD, GAD, SP (DSM-IV-TR)	First-line; Second-line; Third-line; Not recommended	95	Funding from CPA
RANZCP (20)	2003	Australian and New Zealand clinical practice guidelines for the treatment of panic disorder and agoraphobia	Original	Australia & New Zealand	RANZCP	PDA (DSM-IV)	Not reported	17	Funding from the National Mental Health Strategy (Australia)
Bandelow et al. (21)	2022	The German guidelines for the treatment of anxiety disorders: First revision	Updated	Germany	ASMS	PDA, GAD, SAD, SP (ICD-10)	Positive recommendation: Negative recommendation	12	Open Access funding enabled and organized by Projekt DEAL
Asakura et al. (22)	2021	Clinical practice guideline for social anxiety disorder	Original	Japan	JSARD/JSNP	SAD (ICD-11)	GRADE	22	Funding from the Japanese Society of Anxiety and Related Disorders and Japanese Society of Neuropsychopharmacology
NICE (23)	2013	Social anxiety disorder: Recognition, assessment, and treatment	Original	The United Kingdom	NICE	SAD (Not reported)	GRADE	33	Funding from NICE
Katzman et al. (24)	2014	Canadian clinical practice guidelines for the management of anxiety, posttraumatic stress, and obsessive-compulsive disorders	Original	Canada	ADAC	SAD, GAD, PDA, SP, separation anxiety disorder (DSM-IV)	First-line; Second-line; Third-line; Not recommended	83	Funding from CAGIG
HongXiao et al. (25)	2023	Guideline for the diagnosis and treatment of generalized anxiety disorder with integrated Traditional Chinese and Western Medicine	Original	China	Chinese Association of Integrative Medicine/Chinese Association of Chinese Medicine/Chinese Medical Association	GAD (ICD-11 & DSM-V)	EBR & CBR	8	None
QiSheng et al. (26)	2021	International Clinical Practice Guidelines for Traditional Chinese Medicine Anxiety Disorders	Original	China	World Federation of Chinese Medicine Societies/China Association of Chinese Medicine	GAD, PD (DSM-V)	Not reported	4	None
Gautam et al. (27)	2017	Clinical Practice Guidelines for the Management of Generalized Anxiety Disorder and Panic Disorder	Original	India	IPS	GAD, PD (ICD-10)	Not reported	7	None

*CSP–CMA*, Chinese Society of Psychiatry–Chinese Medical Association; *WPSBP*, World Federation of Societies of Biological Psychiatry; *RANZCP*, Royal Australian and New Zealand College of Psychiatrists; *NICE*, National Institute for Health and Clinical Excellence; *MOH*, Ministry of Health, Singapore; *CPA*, Canadian Psychiatric Association; *ASMS*, Association of Scientific Medical Societies (Germany); *JSARD*, Japanese Society of Anxiety and Related Disorders; *JSCP*, Japanese Society of Neuropsychopharmacology; *CAGIG*, Canadian Anxiety Guidelines Initiative Group; *IPS*, Indian Psychiatric Society; *PDA*, Panic disorder and agoraphobia; *GAD*, Generalized anxiety disorder; *SAD*, Social anxiety disorder; *PD*, Panic disorder; *SP*, Specific phobias; *SM*, Selective Mutism; *ICD-10*, International Classification of Diseases (Tenth Edition); *ICD-11*, International Classification of Diseases (Eleventh Edition); *DSM-V*, Diagnostic and Statistical Manual of Mental Disorders (Fifth Edition); *DSM-IV*, Diagnostic and Statistical Manual of Mental Disorders (Fourth Edition); *DSM-IV-TR*, Diagnostic and Statistical Manual of Mental Disorders, Fourth edition, text revision; *EBR*, Evidence-based recommendations; *CBR*, consensus-based recommendation.

### 3.3 Quality of the guidelines

**Supplementary Table 2** presents the AGREE II scores for all 14 guidelines. The average AGREE II scores for the guidelines corresponding to the six domains were: “Scope and Purpose” – 94.1%, “Stakeholder Involvement” – 83.9%, “Rigor of Development” – 77.5%, “Clarity of Presentation” – 92.3%, “Applicability” – 77.9%, and “Editorial Independence” – 70.4%. Ten guidelines from seven organizations, namely WFSBP, RANZCP, National Institute for Health and Care Excellence, Ministry of Health (MOH), Canadian Psychiatric Association (CPA), Association of Scientific Medical Societies (Germany) (ASMA), Japanese Society of Anxiety and Related Disorders (JSARD)/Japanese Society of Neuropsychopharmacology (JSNP), and Canadian Anxiety Guidelines Initiative Group, were regarded as “recommended” (6, 16–24). Four remaining guidelines were scored as “recommended with modifications” (7, 25–27), whereas no guideline was regarded as “non-recommendation.” The ICC value for the assessment results using AGREE II was 0.854, indicating that the internal agreement of the four assessors was relatively consistent. **Supplementary Figure 2** illustrates the overall quality score of AGREE II for each guideline. **Supplementary Figure 3** compares scores across the six domains for every guideline.

### 3.4 Recommendations for psychosocial interventions for adults with anxiety disorder

**Figure 1:**
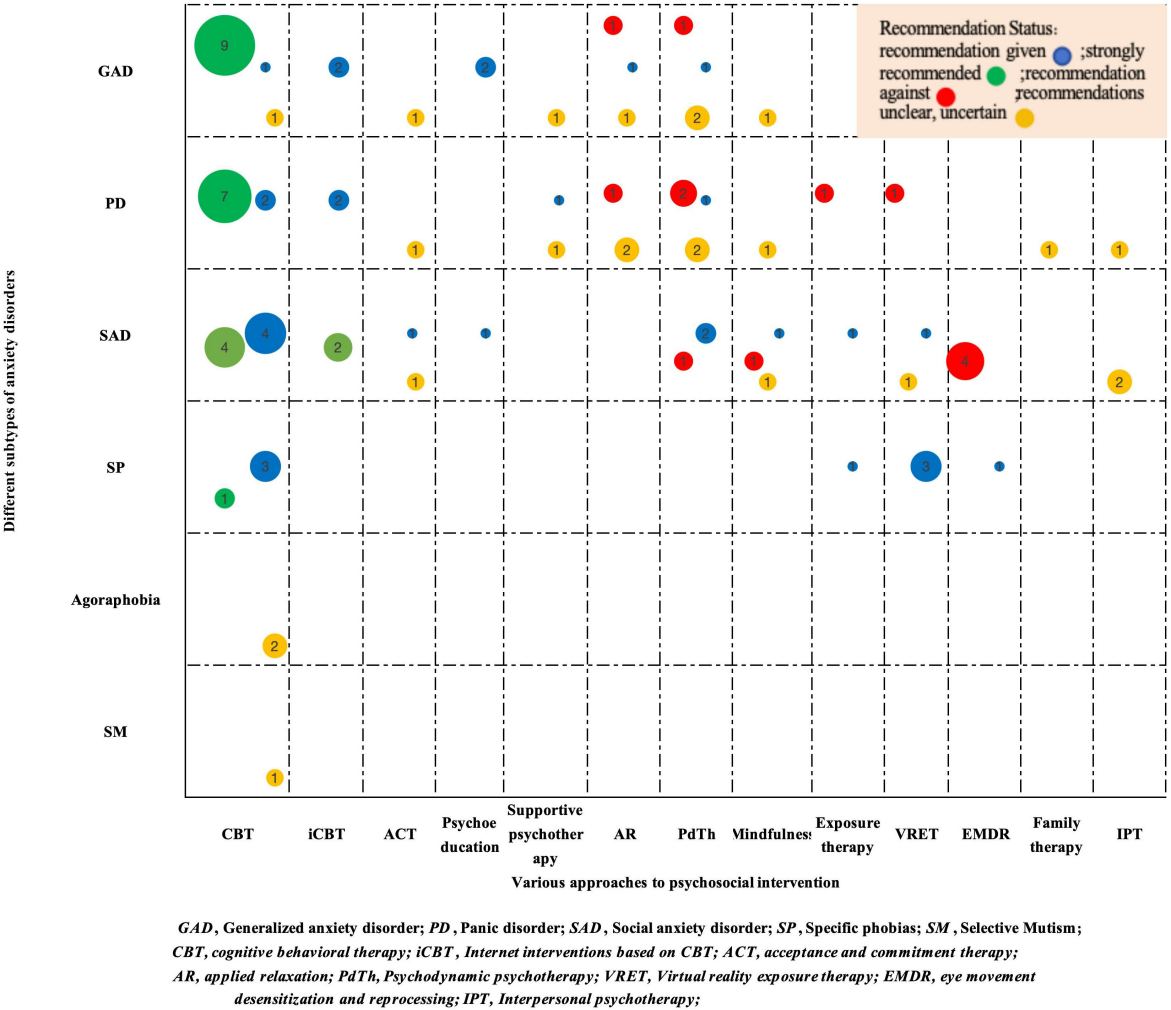
Evidence map of recommended psychosocial interventions in clinical practice guidelines for anxiety disorders.

**Supplementary Table 3** summarizes the recommendations for psychosocial interventions for patients with anxiety in the 14 guidelines. Accordingly, **Figure 1** visualizes and displays the recommended situations of the guidelines. Among them, several recommendations on psychosocial interventions were available for generalized anxiety disorder, panic disorder, and social anxiety disorder. Conversely, recommendations for specific phobias, agoraphobia, and selective mutism were noticeably fewer. During the guideline review, no recommendations for psychosocial interventions for separation anxiety disorder were identified. The WFSBP noted that, since separation anxiety disorder primarily affected children and adolescents, research on separation anxiety disorder in adults was limited; therefore, no recommendations could be made.

For generalized anxiety disorder, nine guidelines strongly recommended CBT as a first-line treatment. Two additional guidelines indicated that internet interventions based on CBT (iCBT) could be recommended to reduce waiting times before initiating face-to-face CBT or as an adjunct to standard CBT. Psychoeducation was recommended for patients with generalized anxiety disorder in two guidelines. Although acceptance and commitment therapy, supportive psychotherapy, and mindfulness were mentioned in the guidelines, these psychosocial interventions were classified as “recommended with uncertainty” or “unclear recommendation” due to insufficient high-quality evidence to validate their effectiveness. The WFSBP did not recommend relaxation or psychodynamic therapy for generalized anxiety disorder, whereas few guidelines included these interventions. Similarly, relaxation and psychodynamic therapy were frequently classified as “recommended with uncertainty” or “unclear recommendation” due to limited evidence.

For panic disorder, seven guidelines strongly recommended CBT as a first-line treatment. Similar to generalized anxiety disorder, two guidelines recommended iCBT as an adjunct to CBT. Few guidelines mentioned supportive psychotherapy, acceptance and commitment therapy, family therapy, interpersonal therapy, and mindfulness; however, these methods were classified as “recommended with uncertainty” or “unclear recommendation” due to limited evidence. There were significant differences in the recommendations for psychodynamic psychotherapy and applied relaxation among the guidelines. Among them, the Association of Scientific Medical Societies recommends psychodynamic psychotherapy as an intervention for panic disorder, RANZCP (16) and Shi and Wenyuan (7) label it as an “unclear recommendation,” and the WFSBP and Canadian Psychiatric Association (CPA) refrain from recommending it. CPA advises against using applied relaxation as an approach for panic disorder, whereas the WFSBP and RANZCP (20) label it as an “unclear recommendation.” Two guidelines do not recommend exposure therapy and virtual reality exposure therapy for panic disorder. The WFSBP, RANZCP (16), CPA, and RANZCP (20) do not recommend eye movement desensitization and reprocessing for managing panic disorder.

For social anxiety disorder, four guidelines strongly recommended CBT as a first-line treatment. The WFSBP and ADAC recommended iCBT as an adjunct to CBT. Separate guidelines recommended psychoeducation and exposure therapy. Currently, the quality of evidence for acceptance and commitment therapy, virtual reality exposure therapy, and interpersonal psychotherapy is inconsistent, leading existing guidelines to avoid clear recommendations or acknowledging their potential benefits. There are differences in recommendations for psychodynamic psychotherapy and mindfulness among various guidelines. Guidelines from Association of Scientific Medical Societies (21) and National Institute for Health and Clinical Excellence (23) recommend psychodynamic psychotherapy for social anxiety disorder, whereas WFSBP did not recommend it. Shi and Wenyuan (7) suggest using mindfulness therapy to treat social anxiety disorder. The recommendation for RANZCP (16) is unclear, whereas the National Institute for Health and Clinical Excellence (23) does not recommend mindfulness as a routine treatment for social anxiety disorder.

For specific phobias, the existing guidelines recommended four psychosocial interventions: CBT, exposure therapy, virtual reality exposure therapy, and eye movement desensitization and reprocessing. Some guidelines suggested CBT for agoraphobia and selective mutism; however, due to inconsistent evidence, CBT was classified as “recommended with uncertainty” or “unclear recommendation.” Currently, no guidelines recommend psychosocial interventions for separation anxiety disorder.

## 4 Discussion

### 4.1 Principal findings

AGREE II evaluation results indicate that the 14 guidelines included in this study were of high quality, with those from JSARD/JSNP (22), RANZCP (16), and MOH (18) ranking among the top performers. Specifically, “Scope and Purpose” and “Clarity of Presentation” attained remarkable scores, reflecting well-defined clinical issues, clear target populations, and unambiguous presentation of recommendations facilitating evidence accessibility for clinicians. Some guidelines have standardized scores of less than 60% in the four domains of “Stakeholder Involvement,” “Rigor of Development,” “Applicability,” and “Editorial Independence.” The rationale is that, in “Stakeholder Involvement,” some guidelines (27) did not include patients as stakeholders in the development process. In “Rigor of Development,” some guidelines (27) did not provide detailed descriptions of the methods for retrieving evidence and the strength of the evidence. In “Applicability,” although some guidelines (25) describe implementation strategies, there is a lack of evidence on potential obstacles in the application. In “Editorial Independence,” during the compilation process, a few guidelines (7, 26) did not elaborate on the conflicts of interest and the project funding details among those involved in developing the guidelines. Thus, when formulating or revising the guidelines for psychosocial interventions for anxiety disorders in the future, involving stakeholders, considering patient preferences and values, and developing recommendations scientifically and rigorously while factoring in the barriers to the clinical implementation of evidence is necessary. This way, the recommendations can be genuinely and effectively utilized in clinical practice.

The evidence map in this report provides an overview of the psychosocial interventions for patients with anxiety disorders. Our evidence mapping analysis revealed that: 1) CBT is supported by robust clinical trial evidence as the first-line psychosocial intervention for generalized anxiety disorder, panic disorder, and social anxiety disorder. 2) Significant evidence gaps exist regarding the efficacy of applied relaxation and psychodynamic psychotherapy for generalized anxiety disorder and panic disorder, necessitating methodologically rigorous clinical trials to establish their therapeutic value. 3) The existing evidence fails to support the use of eye movement desensitization and reprocessing, exposure therapy, or virtual reality exposure therapy in the management of panic disorder. Future research should explore contextual and cultural factors that may influence treatment outcomes. 4) To study the therapeutic efficacy of psychodynamic psychotherapy and interpersonal psychotherapy for social anxiety disorder, mindfulness-based approaches and high-caliber randomized controlled trials are essential. 5) Limited evidence exists for CBT in agoraphobia and SM, with uncertain intervention efficacy requiring further study.

### 4.2 Strengths, limitations, and comparison with other studies

Presently, no systematic review of psychosocial intervention guidelines for adult patients with anxiety has been retrieved. However, during the literature review process, researchers found two related studies (11, 29) on complementary and alternative therapies for patients with anxiety. In the inclusion section of the guidelines, Zhao, Kennedy, Xu, Conduit, Wang, Zhang, Wang, Yue, Huang, Wang, Xu, Fu and Zheng (11) included ten guidelines (published 2003–2022) in their review, whereas Ng and Jain (29) included 11 guidelines (published 2011–2020) in their review. There were 6 overlapping guidelines between this study and the two aforementioned studies (16, 18, 20, 21, 24, 27), accounting for 42.9% of all guidelines included in this study. This finding indicates that the evidence base of this study is largely consistent with the core guideline framework widely recognized in the field. Notably, this study identified 8 unique guidelines that were not included in the two prior reviews. Of these, 2 were newly published between 2021 and 2023 (6, 22), 3 were China-specific guidelines (7, 25, 26), and 3 were previously overlooked guidelines (17, 19, 23). The inclusion of these unique guidelines provides new evidence and perspectives for the present study. In the recommended population section, the article (29) does not provide clear recommendations for specific subtypes of anxiety disorders, and some recommendations are aimed at cancer survivors, breast cancer survivors, and common mental health disorders. In the recommendation section, only a small part of the article mentions psychosocial interventions and only reviews mindfulness and the application of relaxation. Bandelow, Michaelis and Wedekind (5) conducted a systematic study of treatment recommendations for anxiety disorders based on guidelines and only mentioned CBT as the psychosocial intervention.

Compared with previous studies, our review offers the following advantages. We conducted a relatively systematic and comprehensive search of domestic and international guideline websites, professional association websites, and relevant databases, incorporating more and newer guidelines. The entire process, from literature screening to the integration of recommended opinions, was independently conducted by 2–4 researchers, enabling this study to reach comprehensive and reliable conclusions. This study adopted innovative forms such as radar charts and evidence graphs to visually demonstrate the quality and recommended opinions of social psychosocial intervention guidelines for adult anxiety disorders, while further refining the social psychosocial intervention methods applicable or not applicable to different anxiety disorders. In addition, this study identified gaps in existing guideline recommendations, such as the lack of social and psychosocial intervention recommendations for adult dissociative anxiety disorder, uncertainty in recommendations for mindfulness therapy or psychodynamic therapy, which can provide clear guidance for future research.

However, our research has some limitations. First, it only included guidelines in Chinese and English, and did not search for grey literature, which cannot fully cover all recommendations for social and psychosocial interventions for adult anxiety disorder patients worldwide, and may lead to biased results. Second, this study may involve cultural biases, such as differences in the recognition and value of some intervention measures such as mindfulness therapy and psychodynamics across countries. Future research can enhance global applicability through cultural adaptation adjustment or cross-cultural comparative studies.

### 4.3 Implications for guideline updates/developments and clinical applications

To date, many countries have developed clinical practice guidelines for anxiety disorders and have pointed out that psychosocial interventions are an important component of the treatment regimen for anxiety disorders. The WHO Mental Health Action Plan (2023-2030) (30) explicitly proposed to “expand the coverage of mental health services, with particular attention to low - and middle-income countries.” However, the fairness of global medical guidelines still faces significant regional differences, with the “guideline practice gap” resultant from resource constraints in low - and middle-income countries being particularly prominent. For example, only 15% of patients with anxiety disorder in sub Saharan Africa can receive standardized treatment, which is far lower than the 70% in high-income countries (31). Studies (32–34) have indicated that in the past decade, the number of people seeking help for anxiety disorders has increased significantly. However, most of their treatment and care are not evidence-based. A cross-sectional survey (35) on doctors’ implementation of clinical practice guidelines has shown that approximately 30% of doctors believe that the guidelines are too complex, making it difficult to find the necessary information and use it in clinical practice. This suggests that researchers should strictly follow the steps for creating guidelines, such as the development process recommended by the American College of Physicians (36), to support clinicians in providing excellent healthcare and closing the existing gap between what the evidence suggests and what is done in practice. Future research may also consider developing practical guidelines for psychosocial interventions for anxiety disorders, which can be directly used by interest groups. At the same time, low-cost intervention programs—such as developing a visual social and psychosocial intervention training manual to enhance the social and psychosocial intervention capabilities of grassroots medical staff in low - and middle-income countries, and reducing per capita costs through group social and psychosocial intervention models—can be explored to further promote accessible and executable standardized interventions for populations in low - and middle-income countries, to narrow the global gap in health equity in anxiety treatment.

The existing guidelines have cumulatively provided 13 psychosocial interventions for anxiety disorders. In specific clinical practice, the preferences and motivations of patients determine the choice of intervention methods. Single or combined psychosocial intervention measures can be provided according to the patient’s needs. Concurrently, the accessibility, cost, and safety of the treatment should be considered. For example, effective face-to-face CBT requires a large number of professionally trained psychotherapists to implement and manage it, and the global shortage of professional psychotherapists might limit the effectiveness of psychotherapy.

Moreover, a study cited in Williams et al. (37) indicates that merely 10%–50% of patients obtain proper treatment within healthcare systems. The primary causes of this situation include shortages in mental health resources, extended waiting periods, and insufficient awareness regarding available treatment alternatives. With the latest advances in digital technology, there has been significant development and growth in digital interventions for treating anxiety disorders. While digital interventions bring benefits to patients with anxiety disorders, they effectively address gaps in the implementation of clinical guidelines and expand the coverage of evidence-based nursing. Patients who were previously reluctant or unable to undergo psychosocial interventions now have an alternative in the form of a sequence of structured iCBT (38, 39). The MindDoc APP designed by Kuester et al. (40) improves patient compliance and anxiety symptoms as well as quality of life through a lightweight psychosocial intervention module. Additionally, digital interventions enhance cost-efficiency and allow for a personalized approach to meet the unique requirements of each patient (41). Another example is that in some developing countries, medical resources are limited, and the high cost of psychosocial interventions makes it difficult for patients to receive long-term treatment. Therefore, clinical practitioners can provide such patients with less expensive and more accessible interventions, such as supportive psychotherapy and psychoeducation, to improve the patient’s symptoms.

Following clinical practice, psychosocial interventions with strong recommendations should be prioritized; however, the continuous updating and iteration of guidelines will inform change in the quality of evidence. Therefore, interventions currently defined as “unclear recommendations” should not be completely negated in clinical practice. Rigorous randomized controlled trials can be designed in the future to collect evidence on the efficacy of such psychosocial interventions, determine the quality of the evidence, and provide corresponding evidence.

## 5 Conclusions

Specific disparities in the quality of guidelines correlate with psychosocial interventions for anxiety disorders in adults, especially in “Rigor of Development,” “Editorial Independence,” and “Applicability.” Among the different guidelines, there are a few differences in the advice provided regarding psychosocial interventions. Therefore, while formulating or updating guidelines, researchers must strictly adhere to the development process. While providing high-quality evidence for the adult anxiety disorder population, they should ensure that the guidelines are consistent with the emerging evidence. Additionally, future research should focus on discovering the significant impact of specific psychosocial interventions, such as CBT, and pay attention to interventions classified under “unclear recommendations.” Moreover, in clinical practice, scientific and efficient psychosocial interventions should be enforced. This can decrease the difference between the guidelines and practical operations, help those with anxiety disorders improve their emotional experiences, and increase their quality of life.

## Data Availability

The original contributions presented in the study are included in the article/**Supplementary Material**. Further inquiries can be directed to the corresponding author/s.
