# Supplementary material for: Psychosocial interventions for anxiety disorders in adults: evidence mapping and guideline appraisal
Source: Front Psychiatry. 2025 Oct 20;16:1677705. doi: 10.3389/fpsyt.2025.1677705 (PMC12580650; doi:10.3389/fpsyt.2025.1677705)

Supplementary Material

# Supplementary Figures and Tables

## Supplementary Figures


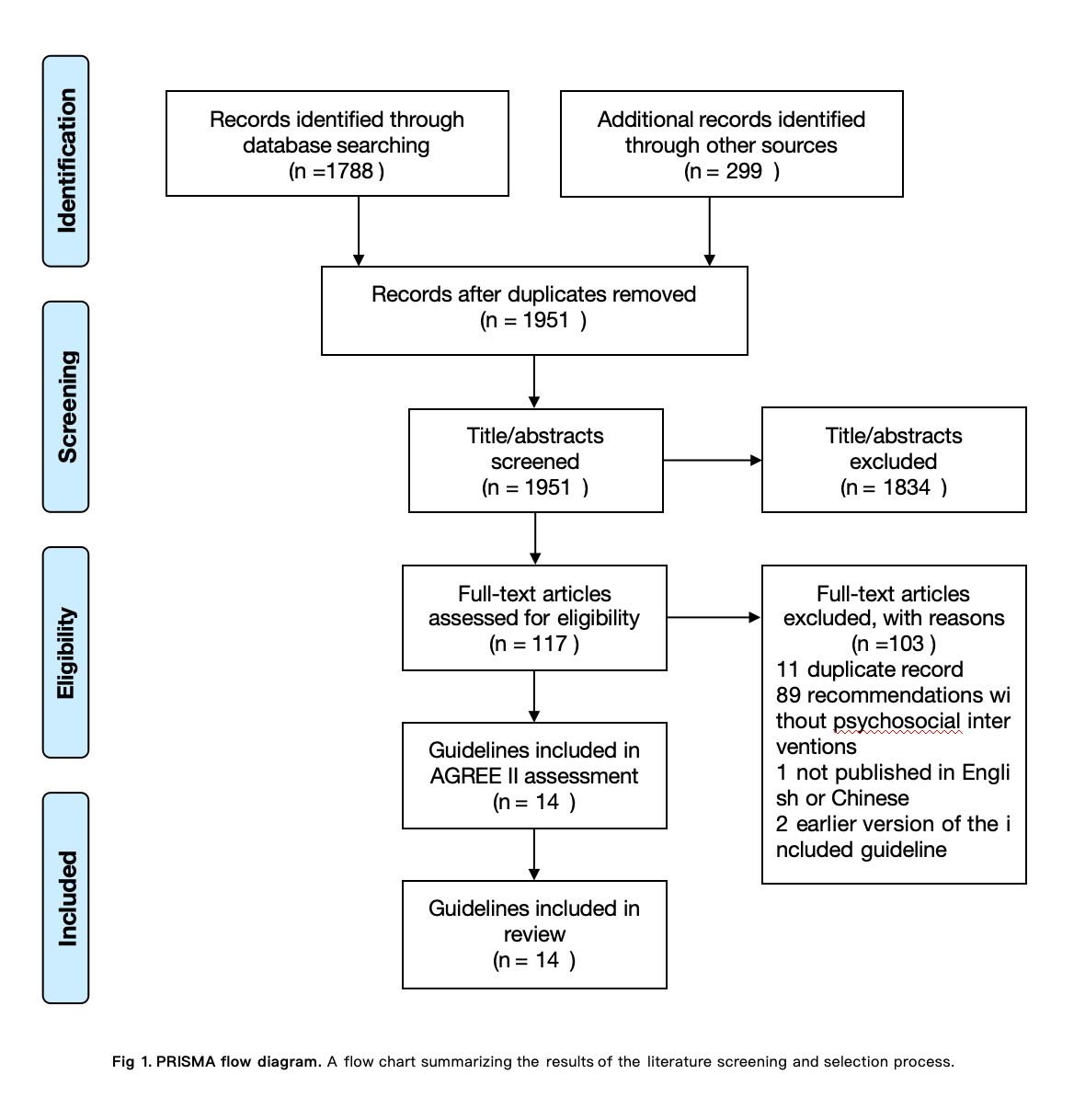


**Supplementary Figure 1.** PRISMA flow diagram


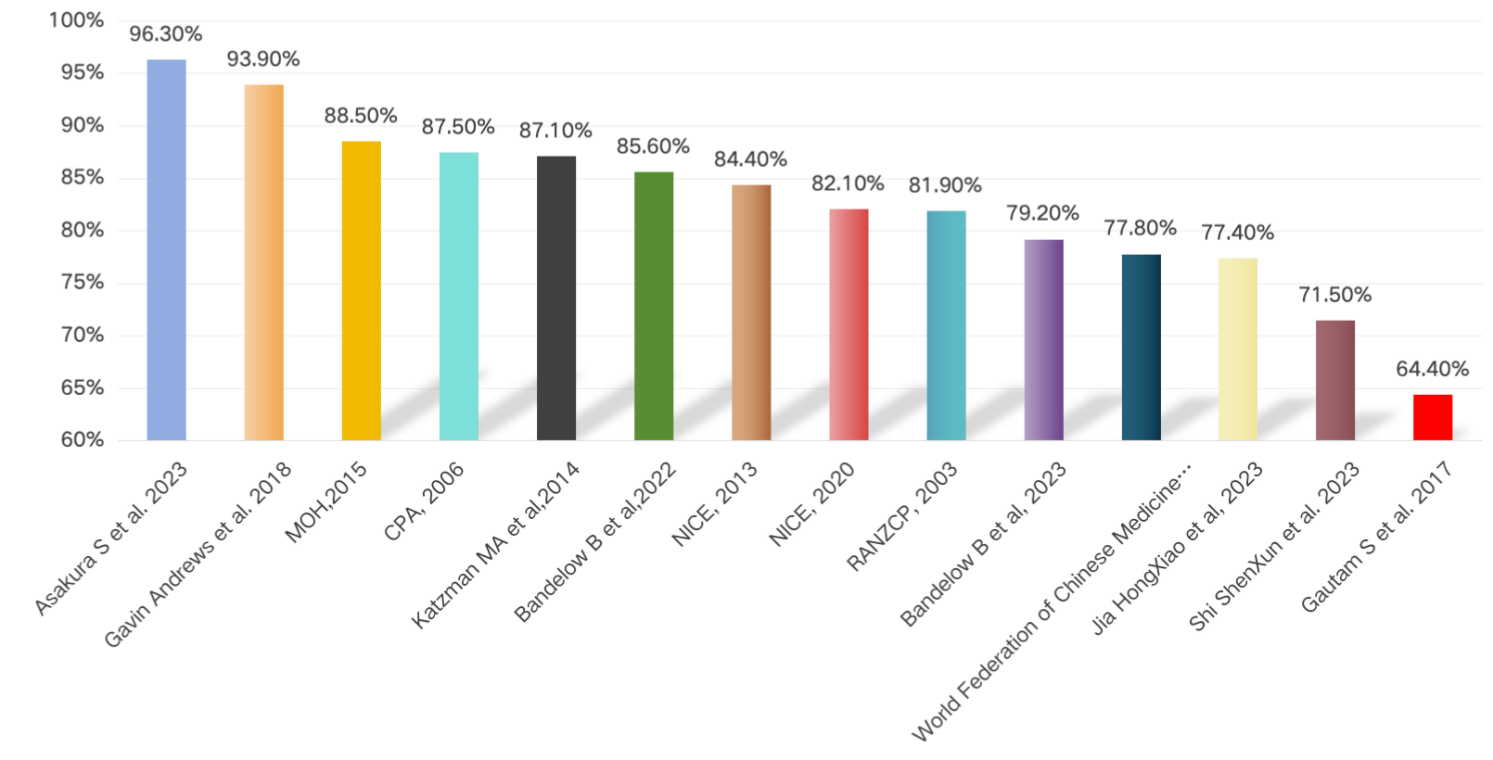


**Supplementary Figure 2.** Overall quality score of AGREE II for the fourteen guidelines


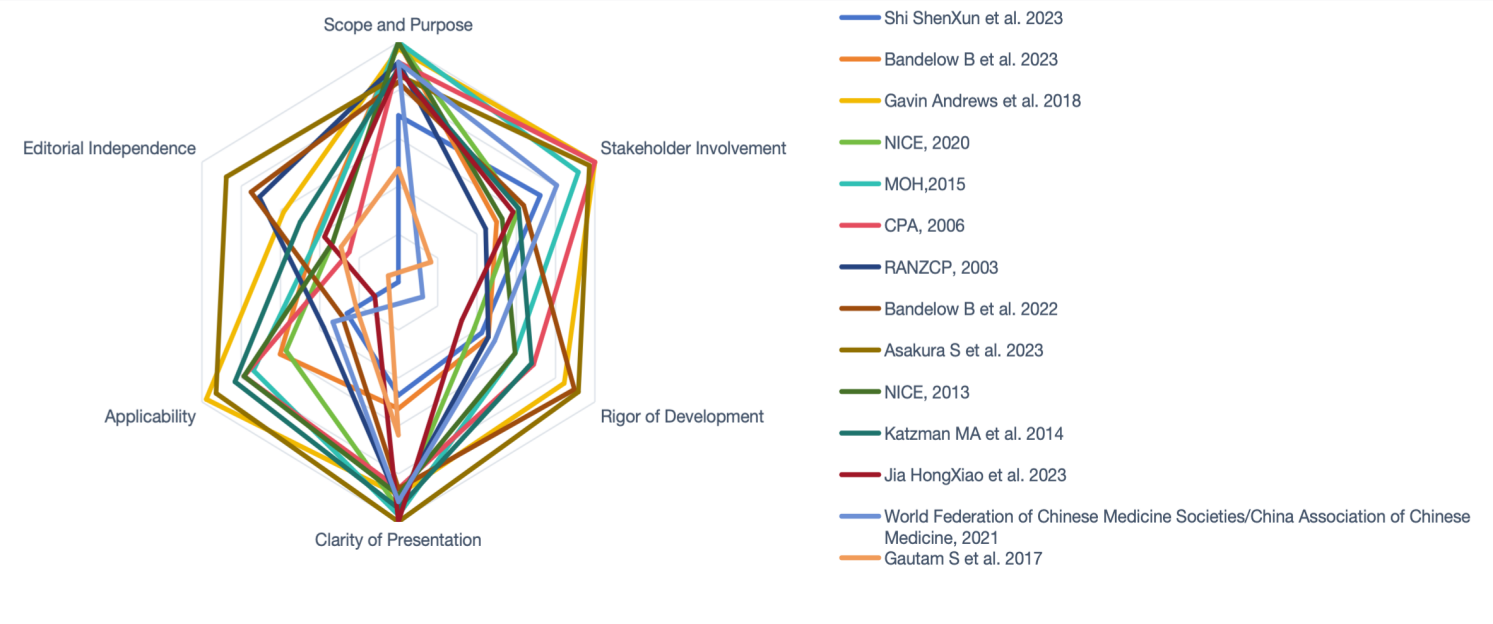


**Supplementary Figure 3.** Scores for six domains in each guideline


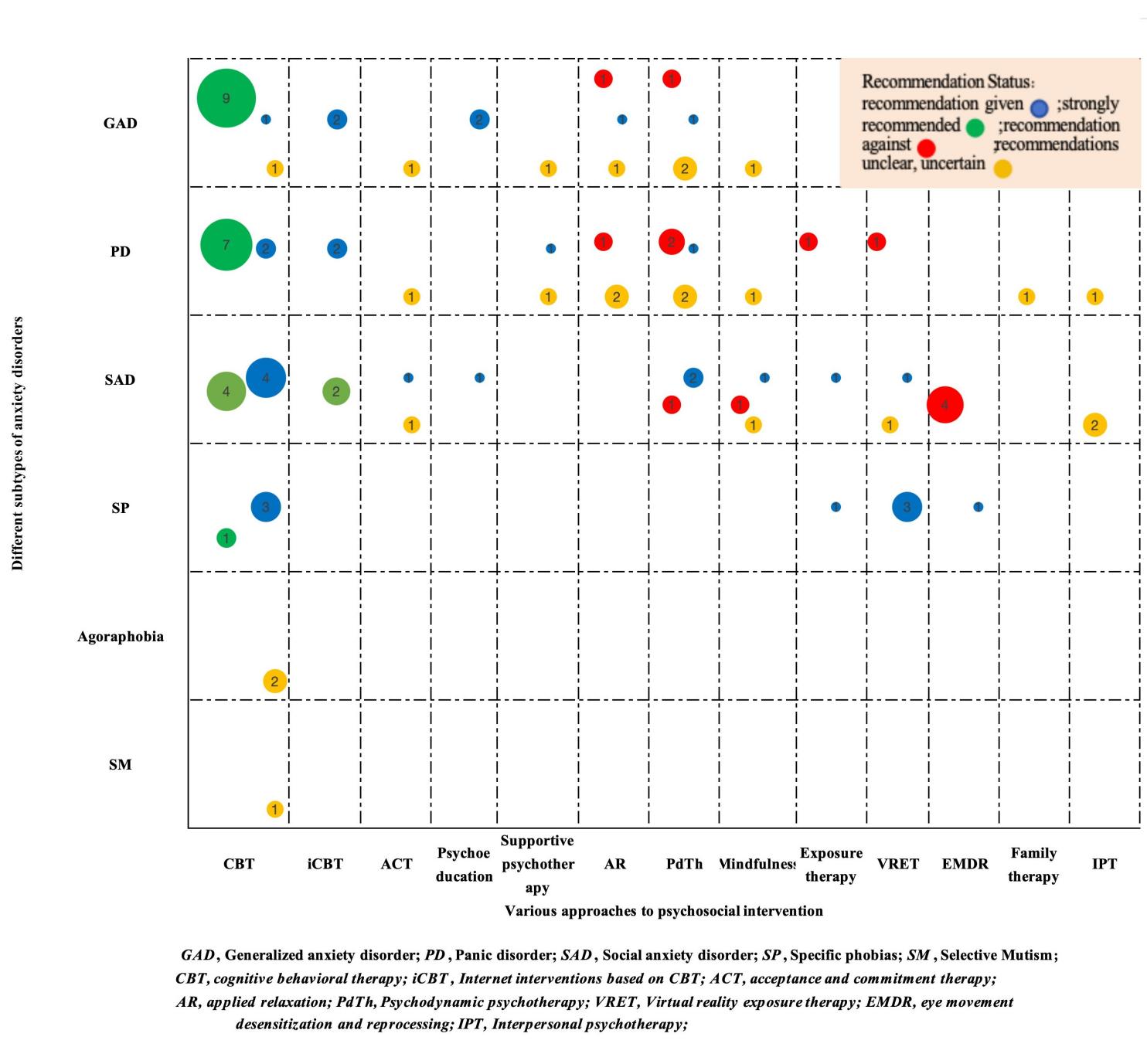


**Supplementary Figure 4.** Evidence map of recommended psychosocial interventions in anxiety disorders clinical practice guidelines

## Supplementary Tables

**Supplementary Table 1.** Characteristics of the clinical practice guidelines.

| **Author** | **Year** | **Guideline theme** | **Version** | **Country/**  **Region** | **Primary developer/ Publishing entity** | **Types of**  **anxiety disorders (Diagnosis)** | **Strength of the**  **recommendations** | **Guideline**  **page** | **Funding** |
| --- | --- | --- | --- | --- | --- | --- | --- | --- | --- |
| S. X.et al. (1) | 2023 | Guidelines for the Prevention and Treatment of Anxiety Disorders in China (Second Edition) | Updated | China | CSP–CMA | GAD, PD, SAD  (ICD-11 & DSM-V) | Not reported | 416 | None |
| Bandelow et al. (2) | 2023 | World Federation of Societies of Biological Psychiatry guidelines for the treatment of anxiety, obsessive-compulsive, and posttraumatic stress disorders-Version 3. Part I: Anxiety disorders | Updated | Argentina | WFSBP | PDA, GAD, SAD, SP, SM  (ICD-10/ICD-11  & DSM-V) | The WFSBP evidence grading system | 39 | None |
| Andrews et al. (3) | 2018 | Royal Australian and New Zealand College of Psychiatrists clinical practice guidelines for the treatment of panic disorder, social anxiety disorder, and generalized anxiety disorder | Original | Australia &  New Zealand | RANZCP | PDA, SAD, GAD  (DSM-V) | EBR & CBR | 64 | Funding from RANZCP |
| NICE (4) | 2020 | Generalized anxiety disorder and panic disorder in adults: Management | Updated | The United Kingdom | NICE | GAD, PD  (DSM-IV & DSM-IV-TR) | GRADE | 47 | Funding from NICE |
| MOH (5) | 2015 | Ministry of Health Clinical Practice Guidelines: Anxiety disorders | Updated | Singapore | MOH | PD, GAD, SP, SAD  (ICD-10 & DSM-IV-TR) | GRADE | 100 | None |
| CPA (6) | 2006 | Management of anxiety disorders | Original | Canada | CPA | PD, SAD, GAD, SP  (DSM-IV-TR) | First-line; Second-line; Third-line; Not recommended | 95 | Funding from CPA |
| RANZCP (7) | 2003 | Australian and New Zealand clinical practice guidelines for the treatment of panic disorder and agoraphobia | Original | Australia &  New Zealand | RANZCP | PDA  (DSM-IV) | Not reported | 17 | Funding from the National Mental Health Strategy (Australia) |
| Bandelow et al. (8) | 2022 | The German guidelines for the treatment of anxiety disorders: First revision | Updated | Germany | ASMS | PDA, GAD, SAD, SP  (ICD-10) | Positive recommendation: Negative recommendation | 12 | Open Access funding enabled and organized by Projekt DEAL |
| Asakura et al. (9) | 2021 | Clinical practice guideline for social anxiety disorder | Original | Japan | JSARD/JSNP | SAD  (ICD-11) | GRADE | 22 | Funding from the Japanese Society of Anxiety and Related  Disorders and Japanese Society of  Neuropsychopharmacology |
| NICE (10) | 2013 | Social anxiety disorder: Recognition, assessment, and treatment | Original | The United Kingdom | NICE | SAD  (Not reported) | GRADE | 33 | Funding from NICE |
| Katzman et al. (11) | 2014 | Canadian clinical practice guidelines for the management of anxiety, posttraumatic stress, and obsessive-compulsive disorders | Original | Canada | ADAC | SAD, GAD, PDA, SP, separation anxiety disorder  (DSM-IV) | First-line; Second-line; Third-line; Not recommended | 83 | Funding from CAGIG |
| HongXiao et al. (12) | 2023 | Guideline for the diagnosis and treatment of generalized anxiety disorder with integrated Traditional Chinese and Western Medicine | Original | China | Chinese Association of Integrative Medicine/Chinese Association of Chinese Medicine/Chinese Medical Association | GAD  (ICD-11 & DSM-V) | EBR & CBR | 8 | None |
| QiSheng et al. (13) | 2021 | International Clinical Practice Guidelines for Traditional Chinese Medicine Anxiety Disorders | Original | China | World Federation of Chinese Medicine Societies/China Association of Chinese Medicine | GAD, PD  (DSM-V) | Not reported | 4 | None |
| Gautam et al. (14) | 2017 | Clinical Practice Guidelines for the Management of Generalized Anxiety Disorder and Panic Disorder | Original | India | IPS | GAD, PD  (ICD-10) | Not reported | 7 | None |

*CSP–CMA*, Chinese Society of Psychiatry–Chinese Medical Association; *WPSBP*, World Federation of Societies of Biological Psychiatry; *RANZCP*, Royal Australian and New Zealand College of Psychiatrists; *NICE*, National Institute for Health and Clinical Excellence; *MOH*, Ministry of Health, Singapore; *CPA*, Canadian Psychiatric Association; *ASMS*, Association of Scientific Medical Societies (Germany); *JSARD*, Japanese Society of Anxiety and Related Disorders; *JSCP*, Japanese Society of Neuropsychopharmacology; *CAGIG*, Canadian Anxiety Guidelines Initiative Group; *IPS*, Indian Psychiatric Society; *PDA*, Panic disorder and agoraphobia; *GAD*, Generalized anxiety disorder; *SAD*, Social anxiety disorder; *PD*, Panic disorder; *SP*, Specific phobias; *SM*, Selective Mutism; *ICD-10*, International Classification of Diseases (Tenth Edition); *ICD-11*, International Classification of Diseases (Eleventh Edition); *DSM-V*, Diagnostic and Statistical Manual of Mental Disorders (Fifth Edition); *DSM-IV*, Diagnostic and Statistical Manual of Mental Disorders (Fourth Edition); *DSM-IV-TR*, Diagnostic and Statistical Manual of Mental Disorders, Fourth edition, text revision; *EBR*, Evidence-based recommendations; *CBR*, consensus-based recommendation.

**Supplementary Table 2.** AGREE II: Appraisal of clinical practice guidelines making recommendations for psychosocial interventions in anxiety disorder.

| **Author, Year of Publication** | **Domain 1: Scope and Purpose** | **Domain 2: Stakeholder Involvement** | **Domain 3: Rigor of Development** | **Domain 4: Clarity of Presentation** | **Domain 5: Applicability** | **Domain 6: Editorial Independence** | **AGREE II Overall Quality Score** | **ICC** | **Recommended Level** |
| --- | --- | --- | --- | --- | --- | --- | --- | --- | --- |
| S. X.et al. (1) | 84.7 | 86.1 | 71.2 | 73.6 | 63.1 | 50.0 | 71.5 | 0.738 | B |
| Bandelow et al. (2) | 100.0 | 75.0 | 72.9 | 76.4 | 80.1 | 70.8 | 79.2 | 0.874 | A |
| Andrews et al. (3) | 98.6 | 100.0 | 92.2 | 94.4 | 98.9 | 79.2 | 93.9 | 0.778 | A |
| NICE (4) | 100.0 | 80.6 | 69.3 | 97.2 | 78.6 | 66.7 | 82.1 | 0.796 | A |
| MOH (5) | 100.0 | 95.8 | 79.7 | 98.6 | 86.9 | 70.1 | 88.5 | 0.861 | A |
| CPA (6) | 95.8 | 100.0 | 84.4 | 93.1 | 89.3 | 62.5 | 87.5 | 0.915 | A |
| RANZCP (7) | 95.8 | 72.2 | 72.9 | 95.8 | 69.0 | 85.4 | 81.9 | 0.841 | A |
| Bandelow et al. (8) | 91.7 | 81.9 | 94.8 | 93.1 | 64.3 | 87.5 | 85.6 | 0.864 | A |
| Asakura et al. (9) | 93.1 | 98.6 | 95.8 | 100.0 | 96.4 | 93.8 | 96.3 | 0.818 | A |
| NICE (10) | 100.0 | 76.4 | 79.7 | 94.4 | 89.3 | 66.7 | 84.4 | 0.886 | A |
| Katzman et al. (11) | 94.4 | 80.6 | 83.9 | 97.2 | 91.6 | 75.0 | 87.1 | 0.879 | A |
| HongXiao et al. (12) | 94.4 | 79.2 | 66.1 | 100.0 | 56.0 | 68.8 | 77.4 | 0.908 | B |
| QiSheng et al. (13) | 95.8 | 90.3 | 74.5 | 95.8 | 66.7 | 43.8 | 77.8 | 0.881 | B |
| Gautam et al. (14) | 73.6 | 58.3 | 47.4 | 81.9 | 60.7 | 64.6 | 64.4 | 0.910 | B |
| Average | 94.1 | 83.9 | 77.5 | 92.3 | 77.9 | 70.4 | 82.7 | 0.854 | — |

**Supplementary Table 3.** Summary of recommendations on psychosocial interventions for adults with anxiety disorder.

|  | G1 | G2 | G3 | G4 | G5 | G6 | G7 | G8 | G9 | G10 | G11 | G12 | G13 | G14 |
| --- | --- | --- | --- | --- | --- | --- | --- | --- | --- | --- | --- | --- | --- | --- |
| GAD | | | | | | | | | | | | | | |
| CBT | (+) | + | (+) | (+) | (+) | (+) | NR | (+) | NR | NR | (+) | (+) | △ | (+) |
| iCBT | NR | + | NR | NR | NR | NR | NR | + | NR | NR | NR | NR | NR | NR |
| ACT | NR | NR | △ | NR | NR | NR | NR | NR | NR | NR | NR | NR | NR | NR |
| Psychoeducation | NR | NR | + | + | NR | NR | NR | NR | NR | NR | NR | NR | NR | NR |
| Supportive psychotherapy | NR | NR | NR | NR | NR | NR | NR | NR | NR | NR | NR | NR | △ | NR |
| AR | NR | − | △ | + | NR | NR | NR | NR | NR | NR | NR | NR | NR | NR |
| PdTh | NR | − | △ | NR | NR | NR | NR | NR | + | NR | △ | NR | NR | NR |
| Mindfulness | NR | NR | △ | NR | NR | NR | NR | NR | NR | NR | NR | NR | NR | △ |
| PD | | | | | | | | | | | | | | |
| CBT | (+) | + | (+) | + | (+) | (+) | (+) | (+) | NR | NR | (+) | NR | NR | NR |
| iCBT | NR | + | NR | NR | NR | NR | NR | + | NR | NR | NR | NR | NR | NR |
| ACT | NR | NR | △ | NR | NR | NR | NR | NR | NR | NR | NR | NR | NR | NR |
| Exposure therapy | NR | NR | NR | NR | NR | − | NR | NR | NR | NR | NR | NR | NR | NR |
| VRET | NR | NR | NR | NR | NR | NR | NR | − | NR | NR | NR | NR | NR | NR |
| Supportive psychotherapy | △ | NR | NR | NR | NR | NR | + | NR | NR | NR | NR | NR | NR | NR |
| EMDR | NR | − | − | NR | NR | − | − | NR | NR | NR | NR | NR | NR | NR |
| AR | NR | △ | NR | NR | NR | − | △ | NR | NR | NR | NR | NR | NR | NR |
| PdTh | △ | − | △ | NR | NR | − | NR | + | NR | NR | NR | NR | NR | NR |
| Mindfulness | NR | NR | NR | NR | NR | NR | NR | NR | NR | NR | NR | NR | NR | △ |
| Family therapy | △ | NR | NR | NR | NR | NR | NR | NR | NR | NR | NR | NR | NR | NR |
| IPT | △ | NR | NR | NR | NR | NR | NR | NR | NR | NR | NR | NR | NR | NR |
| SAD | | | | | | | | | | | | | | |
| CBT | (+) | + | (+) | NR | (+) | + | NR | NR | + | + | (+) | NR | NR | NR |
| iCBT | NR | + | NR | NR | NR | NR | NR | NR | NR | NR | + | NR | NR | NR |
| ACT | NR | + | NR | NR | NR | NR | NR | NR | △ | NR | NR | NR | NR | NR |
| Exposure therapy | NR | NR | NR | NR | NR | + | NR | NR | NR | NR | NR | NR | NR | NR |
| VRET | NR | △ | NR | NR | NR | NR | NR | + | NR | NR | NR | NR | NR | NR |
| Psychoeducation | NR | NR | + | NR | NR | NR | NR | NR | NR | NR | NR | NR | NR | NR |
| PdTh | NR | − | NR | NR | NR | NR | NR | + | NR | + | NR | NR | NR | NR |
| Mindfulness | + | NR | △ | NR | NR | NR | NR | NR | NR | − | NR | NR | NR | NR |
| IPT | △ | NR | △ | NR | NR | NR | NR | NR | NR | NR | NR | NR | NR | NR |
| SP | | | | | | | | | | | | | | |
| CBT | NR | + | NR | NR | (+) | NR | NR | + | NR | NR | + | NR | NR | NR |
| Exposure therapy | NR | (+) | NR | NR | NR | + | NR | NR | NR | NR | (+) | NR | NR | NR |
| VRET | NR | + | NR | NR | NR | + | NR | + | NR | NR | NR | NR | NR | NR |
| EMDR | NR | + | NR | NR | NR | NR | NR | NR | NR | NR | NR | NR | NR | NR |
| Agoraphobia | | | | | | | | | | | | | | |
| CBT | NR | NR | NR | NR | NR | NR | NR | NR | NR | NR | NR | NR | △ | △ |
| Separation anxiety disorder: No recommendations for social psychological intervention | | | | | | | | | | | | | | |
| SM | | | | | | | | | | | | | | |
| CBT | NR | NR | NR | NR | NR | NR | NR | NR | NR | NR | NR | NR | NR | △ |

*G1*, S. X. et al. (1); *G2*, Bandelow et al. (2); *G3*, Andrews et al. (3); *G4*, NICE (4); *G5*, Lim et al. (5); *G6*, Canadian Psychiatric Association (6); *G7*, RANZCP (7); *G8,* Bandelow et al. (8); *G9*, Asakura et al. (9); *G10*, NICE (10); *G11,* Katzman et al. (11); *G12*, HongXiao et al. (12); *G13*, *QiSheng et al.* (13); *G14*, Gautam et al. (14); *GAD*, Generalized anxiety disorder; *PD*, Panic disorder; *SAD*, Social anxiety disorder; *SP*, Specific phobias; *SM*, Selective Mutism; *CBT*, cognitive behavioral therapy; *iCBT*, Internet interventions based on CBT; *ACT*, acceptance and commitment therapy; *AR*, applied relaxation; *PdTh*, Psychodynamic psychotherapy; VRET, Virtual reality exposure therapy; EMDR, eye movement desensitization and reprocessing; *IPT*, Interpersonal psychotherapy;

+/blue = recommendation given; (+)/green = strongly recommended; −/red = recommendation against; △/yellow = recommendations unclear, uncertain; NR/grey = no recommendation reported

**References**

1. Shi SX, Wenyuan Wu. Guidelines for the Prevention and Treatment of Anxiety Disorders in China: 2nd ed. Medical Electronic Audiovisual Publishing House (2023).

2. Bandelow B, Allgulander C, Baldwin DS, Costa DLDC, Denys D, Dilbaz N, et al. World Federation of Societies of Biological Psychiatry (WFSBP) guidelines for treatment of anxiety, obsessive-compulsive and posttraumatic stress disorders – Version 3. part I: Anxiety disorders. World J Biol Psychiatry (2023) 24:(79–117). doi: [10.1080/15622975.2022.2086295](https://doi.org/10.1080/15622975.2022.2086295).

3. Andrews G, Bell C, Boyce P, Gale C, Lampe L, Marwat O, et al. Royal Australian and New Zealand College of Psychiatrists clinical practice guidelines for the treatment of panic disorder, social anxiety disorder and generalised anxiety disorder. Aust N Z J Psychiatry (2018) 52:1109–72. doi: [10.1177/0004867418799453](https://doi.org/10.1177/0004867418799453).

4. National Institute for Health and Clinical Excellence. Generalised anxiety disorder and panic disorder in adults. Management[EB/OL]. https://www.nice.org.uk/guidance/cg113 (2020/06/15b).

5. Ministry of Health (7/14/2015).Anxiety Disorders [Accessed on 1/2025]. http://www.moh.gov.sg/cpg.

6. Canadian Psychiatric Association. Clinical practice guidelines. Management of anxiety disorders. Can J Psychiatry (2006) 51(8)(Suppl. 2):9S–91S.

7. Royal Australian and New Zealand College of Psychiatrists Clinical Practice Guidelines Team for Panic Disorder and Agoraphobia. Australian and New Zealand clinical practice guidelines for the treatment of panic disorder and agoraphobia. Aust N Z J Psychiatry (2003) 37:641–56. doi: [10.1080/j.1440-1614.2003.01254.x](https://doi.org/10.1080/j.1440-1614.2003.01254.x).

8. Bandelow B, Werner AM, Kopp I, Rudolf S, Wiltink J, Beutel ME. The German Guidelines for the treatment of anxiety disorders: First revision. Eur Arch Psychiatry Clin Neurosci (2022) 272:571–82. doi: [10.1007/s00406-021-01324-1](https://doi.org/10.1007/s00406-021-01324-1).

9. Asakura S, Yoshinaga N, Yamada H, Fujii Y, Mitsui N, Kanai Y, et al. Japanese Society of Anxiety and Related Disorders/Japanese Society of Neuropsychopharmacology: Clinical practice guideline for social anxiety disorder (2021). Neuropsychopharmacol Rep (2023) 43:288–309. doi: [10.1002/npr2.12365](https://doi.org/10.1002/npr2.12365).

10. National Institute for Health and Clinical Excellence (2013/05/22a). Social anxiety disorder: Recognition, assessment and treatment. [Accessed on 7/14/2025]. https://www.nice.org.uk/guidance/cg159.

11. Katzman MA, Bleau P, Blier P, Chokka P, Kjernisted K, Van Ameringen M et al. Canadian clinical practice guidelines for the management of anxiety, posttraumatic stress and obsessive-compulsive disorders. BMC Psychiatry (2014) 14(Suppl. 1)(Suppl. 1):S1. doi: [10.1186/1471-244X-14-S1-S1](https://doi.org/10.1186/1471-244x-14-s1-s1).

12. HongXiao J, Xue L, DongQing Y, Zhiyuan H, QunSong W, Jian W, et al. Guideline for the diagnosis and treatment of generalized anxiety disorder with integrated traditional Chinese and western Medicine. Mod Chin Clin Med (2023) 30:21–7 + 35.

13. QiSheng T, Miao Q, WenJun S. International clinical practice guidelines for traditional Chinese medicine anxiety disorders. World Chin Med (2021) 16:1188–91.

14. Gautam S, Jain A, Gautam M, Vahia VN, Gautam A. Clinical practice guidelines for the management of generalised anxiety disorder (GAD) and panic disorder (PD). Indian J Psychiatry (2017) 59(Suppl. 1):S67–73. doi: [10.4103/0019-5545.196975](https://doi.org/10.4103/0019-5545.196975).

**2 List of Different Reports from the Same Study**

**Study 1:**

Lim L, Chan HN, Chew PH, Chua SM, Ho C, Kwek SKD, et al. Ministry of Health clinical

practice guidelines: Anxiety disorders. Singapore Med J (2015) 56:310–5; quiz 316. doi: [10.11622/smedj.2015088](https://doi.org/10.11622/smedj.2015088).

**Study 2:**

Ministry of Health (7/14/2015).Anxiety Disorders [Accessed on 1/2025]. http://www.moh.gov.sg/cpg.

Both Study 1 and Study 2 are guidelines for anxiety disorders published by the Singapore Ministry of Health. However, Study 2 reproduces the introduction and executive summary (including guidelines recommendations) of Study 1 for reference by readers of the Singapore Medical Journal. Study 2 also states that the chapter numbers and page numbers mentioned in the article correspond to the complete version of the guidelines.

Therefore, when extracting recommendations related to adult anxiety disorders, priority should be given to Study 2.

**3 Full search strategies for all databases**

**3.1 Domestic and international guideline websites**

3.1.1 UpToDate

Search Strategy: anxiety (31)


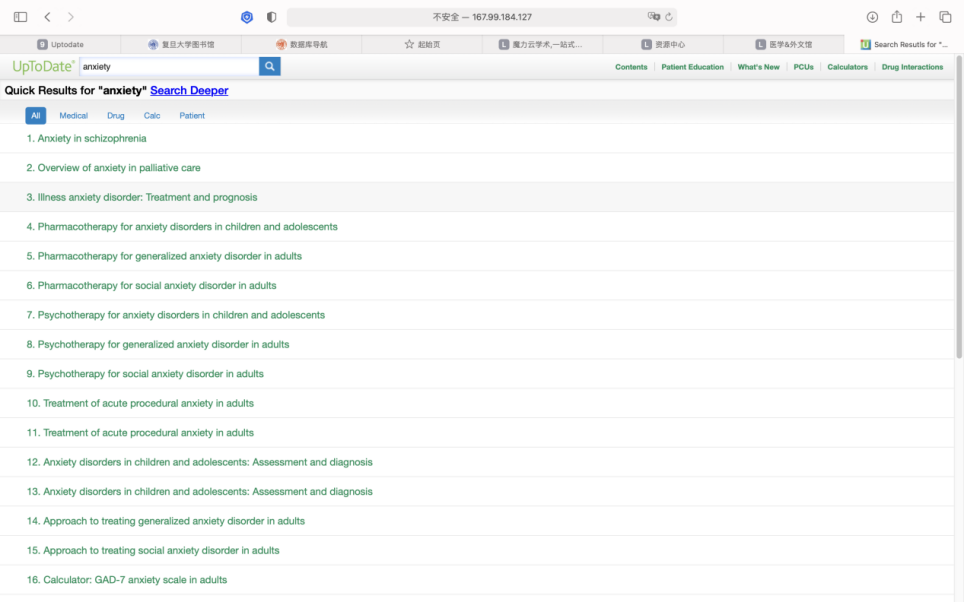


**3.1.2 BMJ Best Practice**

**Search Strategy:** anxiety disorder (3)


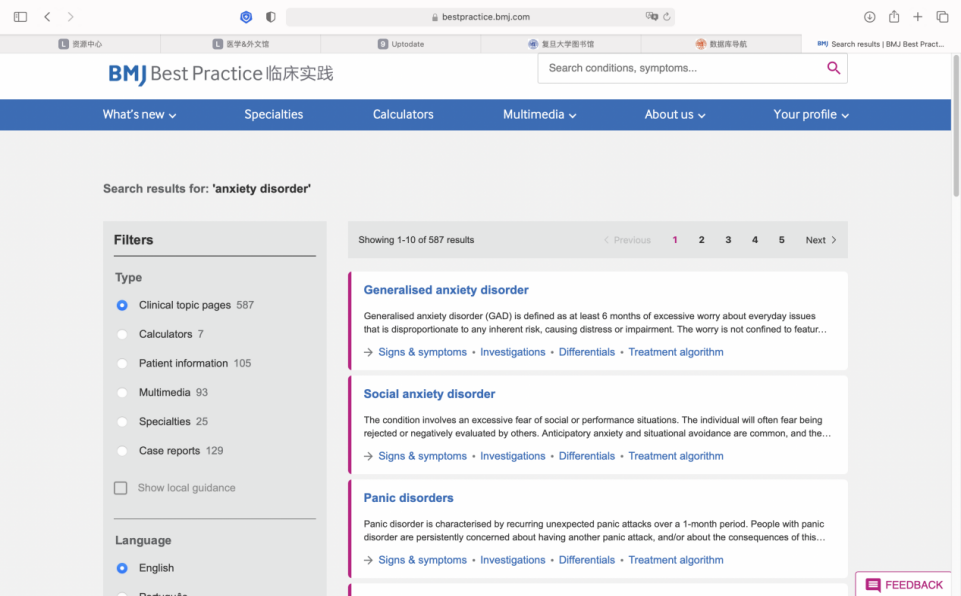


**3.1.3 Guidelines International Network**

**Search Strategy:** anxiety disorder (0)


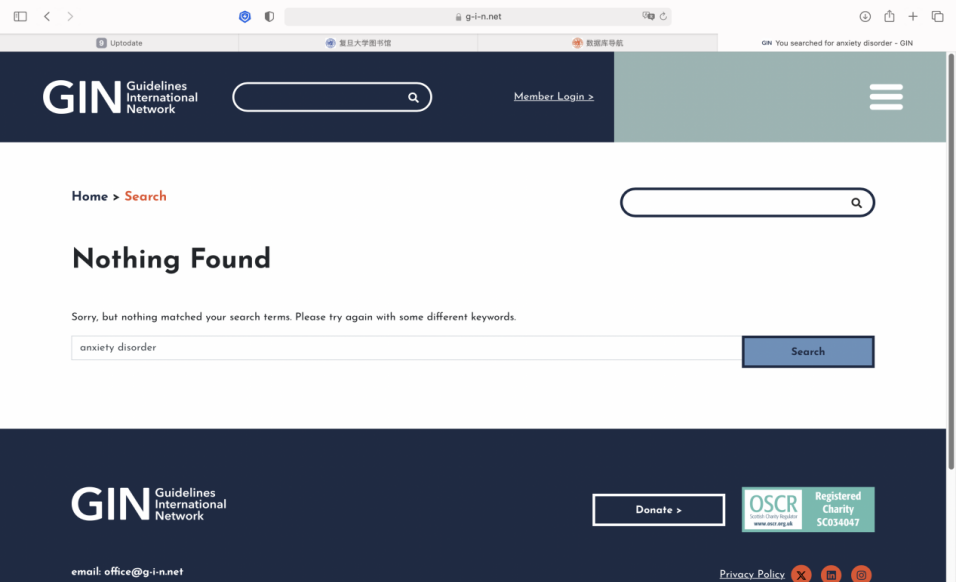


**3.1.4 National Institute for Health and Care Excellence**

**Search Strategy:** anxiety disorder (36)


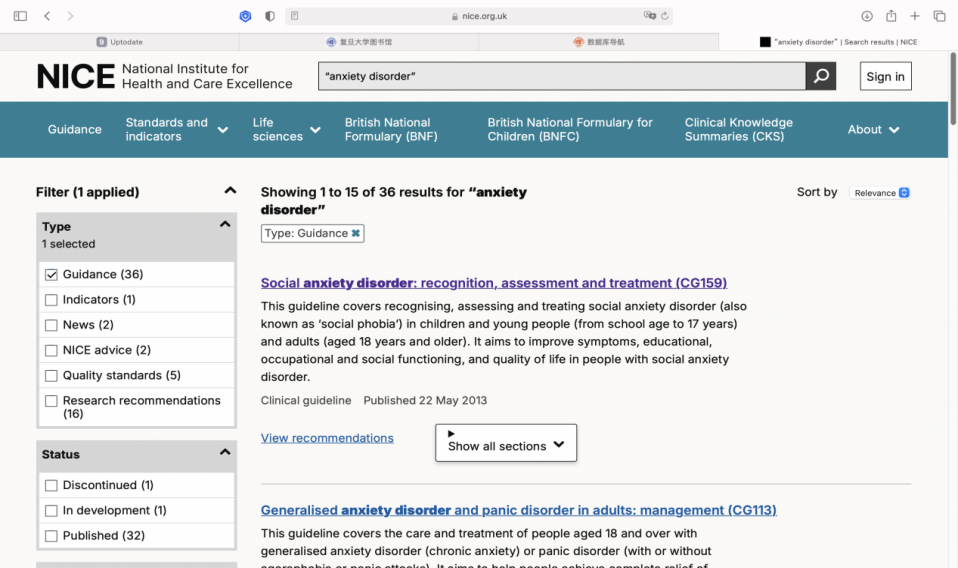


**3.1.5 Scottish Intercollegiate Guidelines Network**

**Search Strategy:** anxiety disorder (0)

**
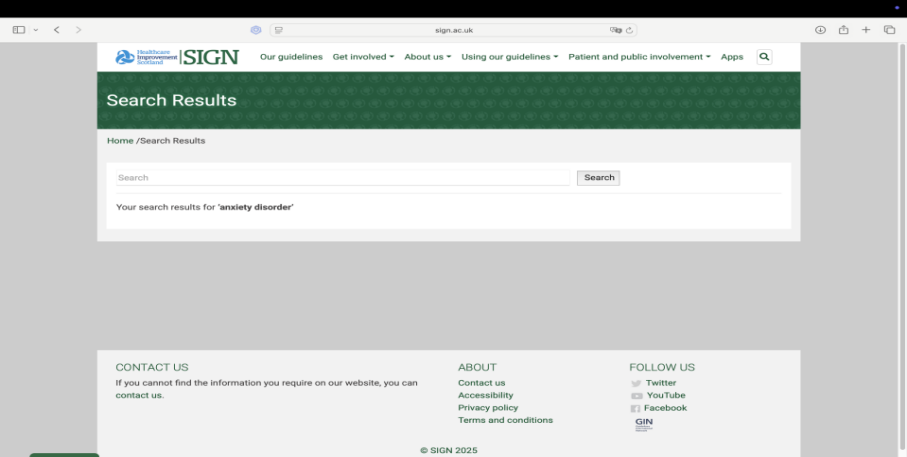
**

**3.1.6 New Zealand Guidelines Group**

**Search Strategy:** anxiety disorder (11)


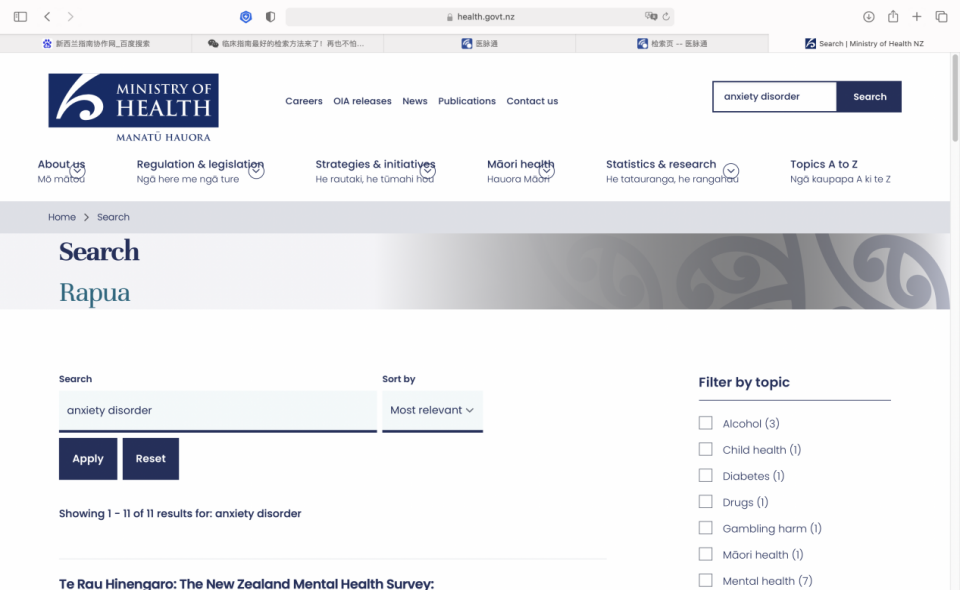


**3.1.7 Agency for Healthcare Research and Quality**

**Search Strategy:** anxiety (27)


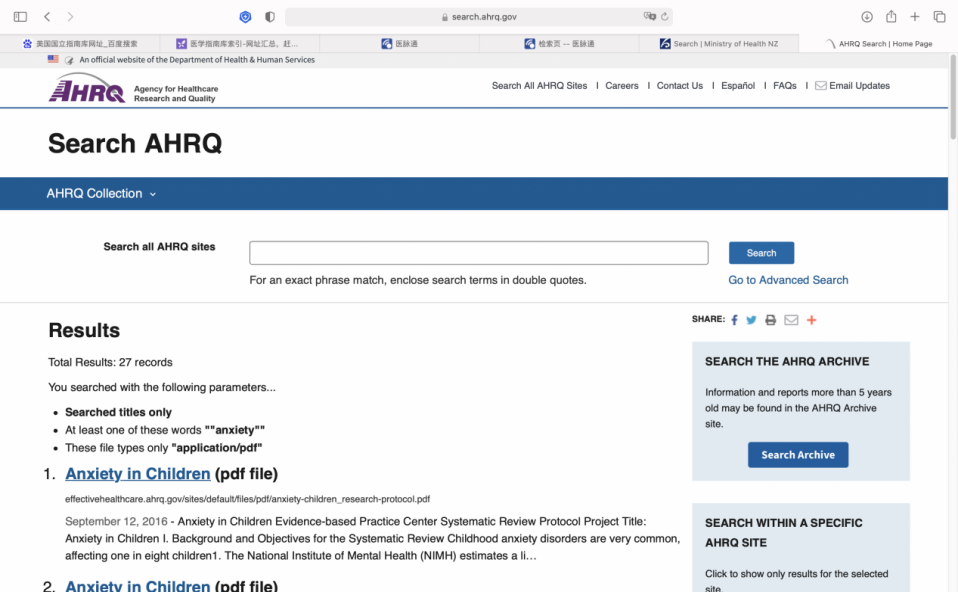


**3.1.8 Registered Nurses’ Association of Ontario**

**Search Strategy:** anxiety disorder (0)


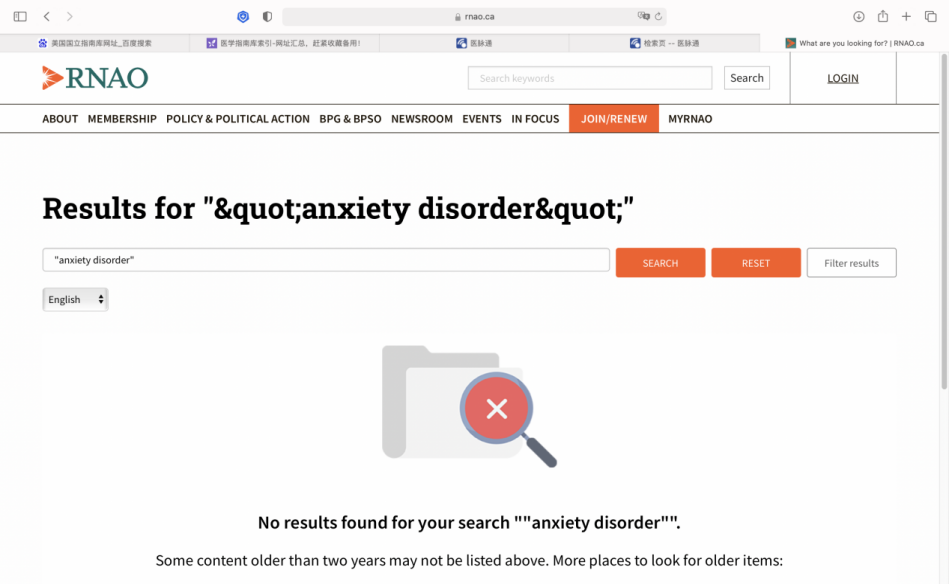


**3.1.9 Medsci Guidelines**

**Search Strategy:** anxiety (177)


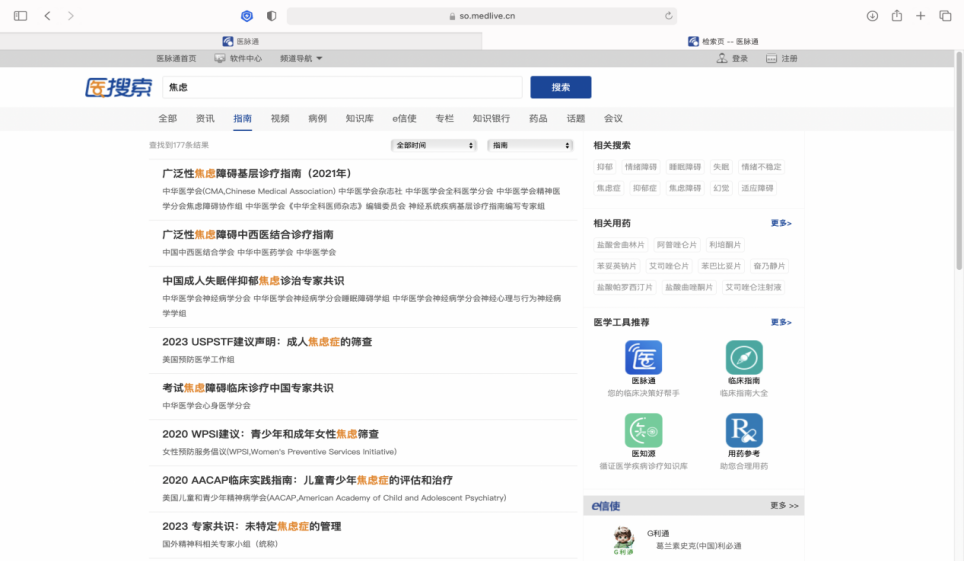


**3.2 Professional association websites**

**3.2.1 American Psychiatric Association**

**Search Strategy:** anxiety disorder (0)


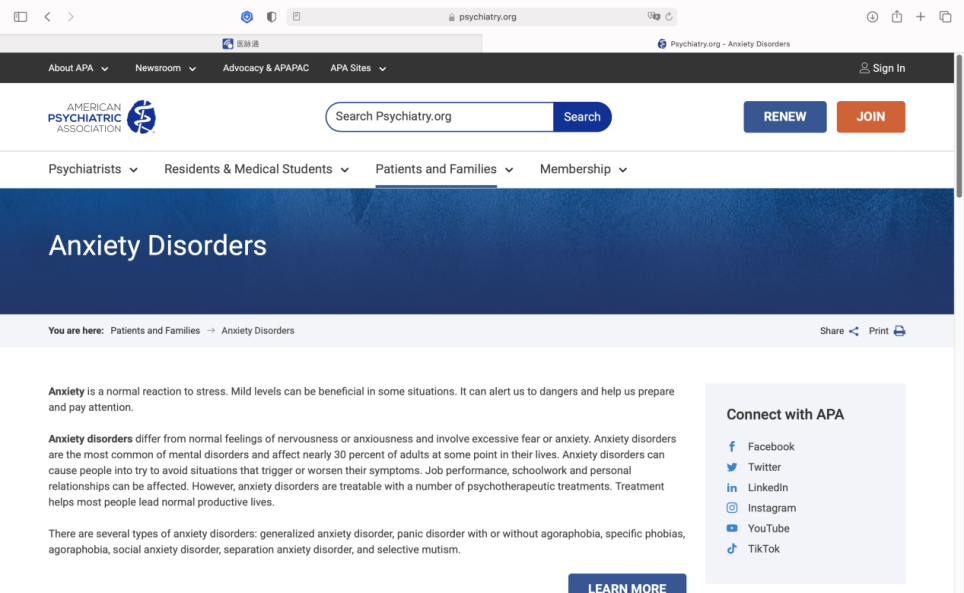


**3.2.2 Anxiety and Depression Association of America**

**Search Strategy:** anxiety disorder (15)


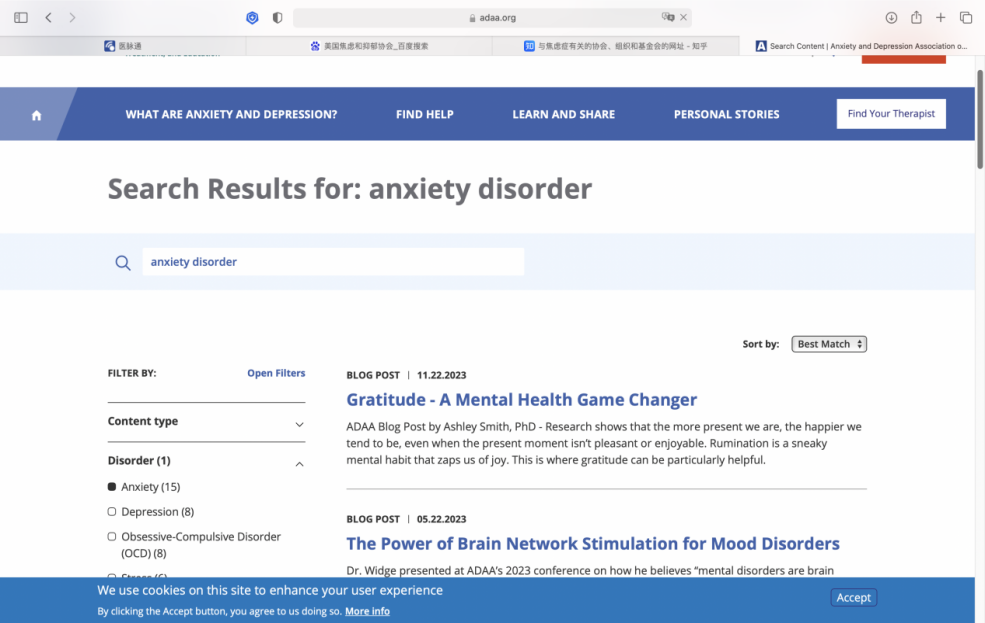


**3.2.3 United States Preventive Services Task Force**

**Search Strategy:** anxiety disorder (17)


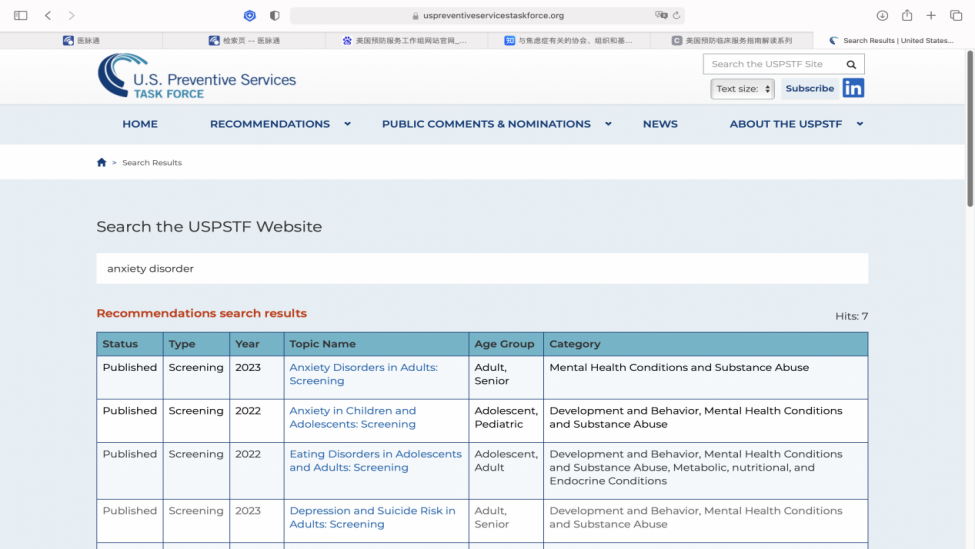


**3.2.4 Royal Australian and New Zealand College of Psychiatrists**

**Search Strategy:** anxiety disorder (7)

**
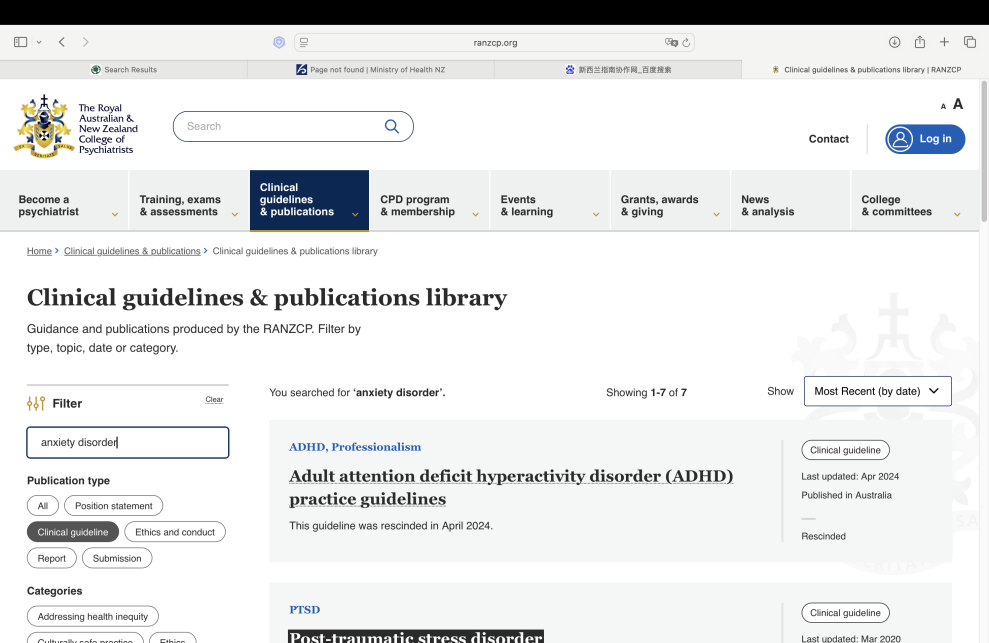
**

**3.2.5 NSW Ministry of Health**

**Search Strategy:** anxiety disorder (0)

**
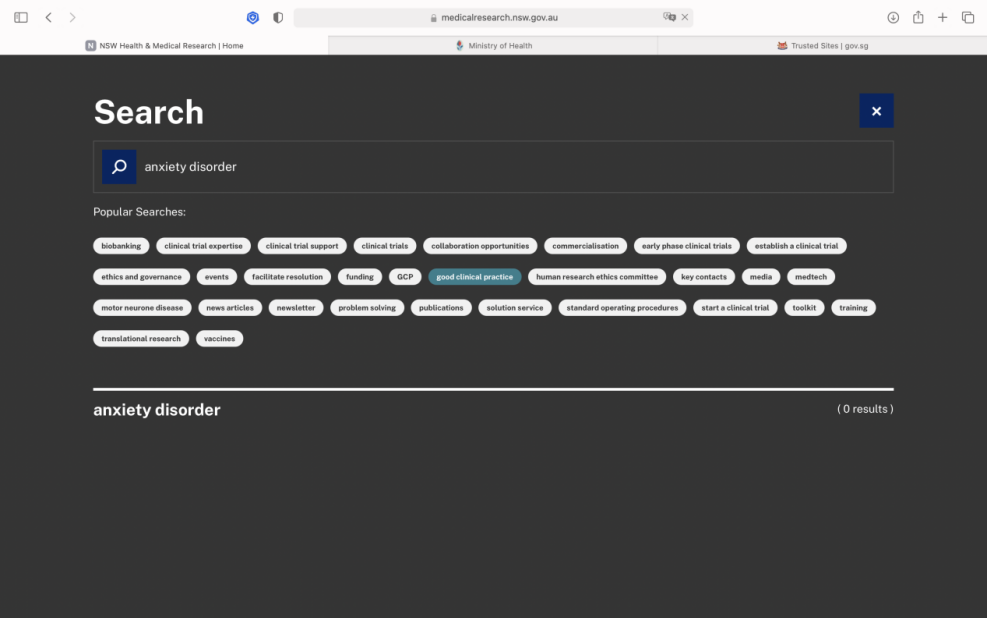
**

**3.2.6 Australian Department of Health and Aged Care**

**Search Strategy:** anxiety disorder (2)


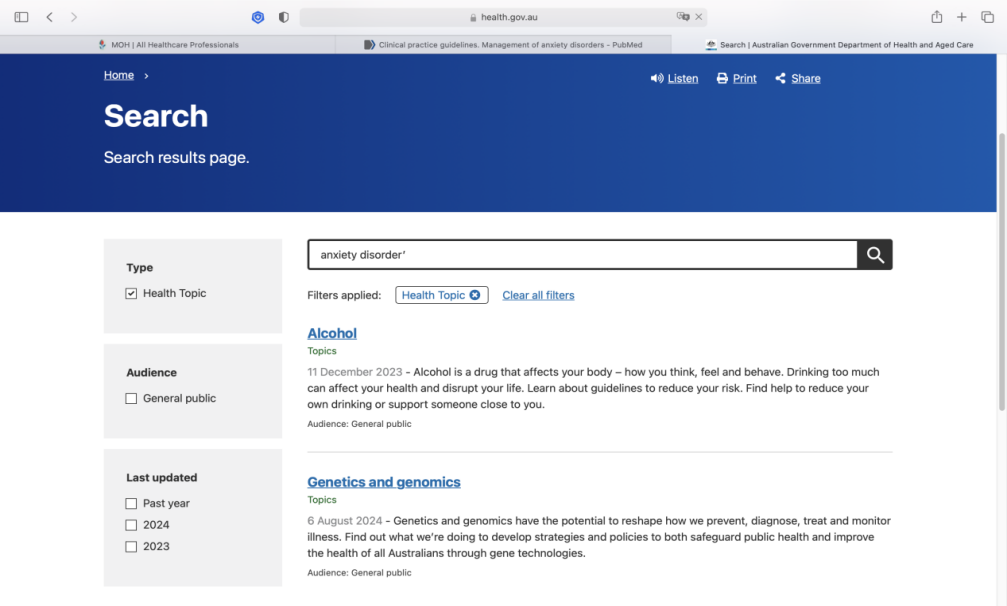


**3.2.7 World Federation of Societies of Biological Psychiatry**

**Search Strategy:** anxiety disorder (2)


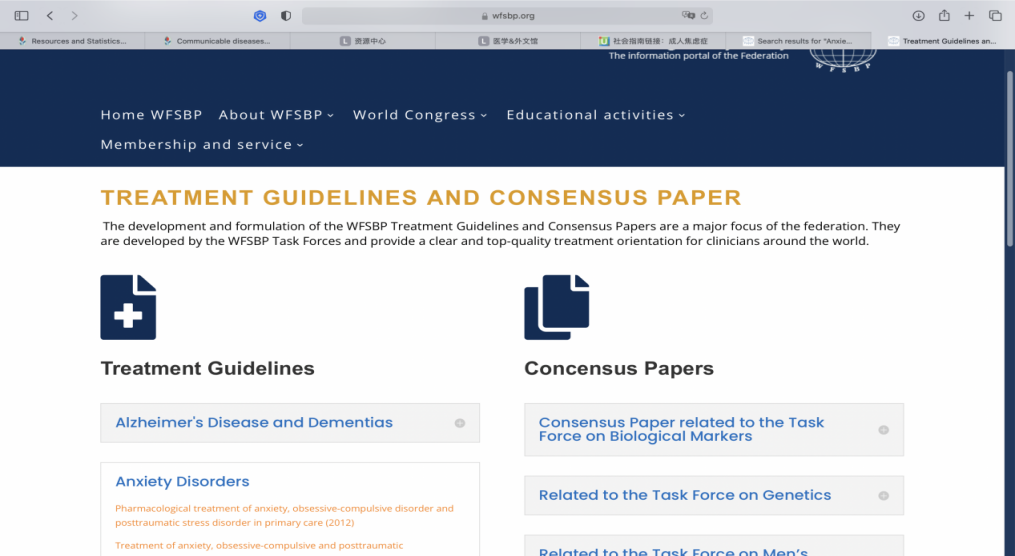


**3.2.8 Chinese Medical Association**

**Search Strategy:** anxiety disorder (4)


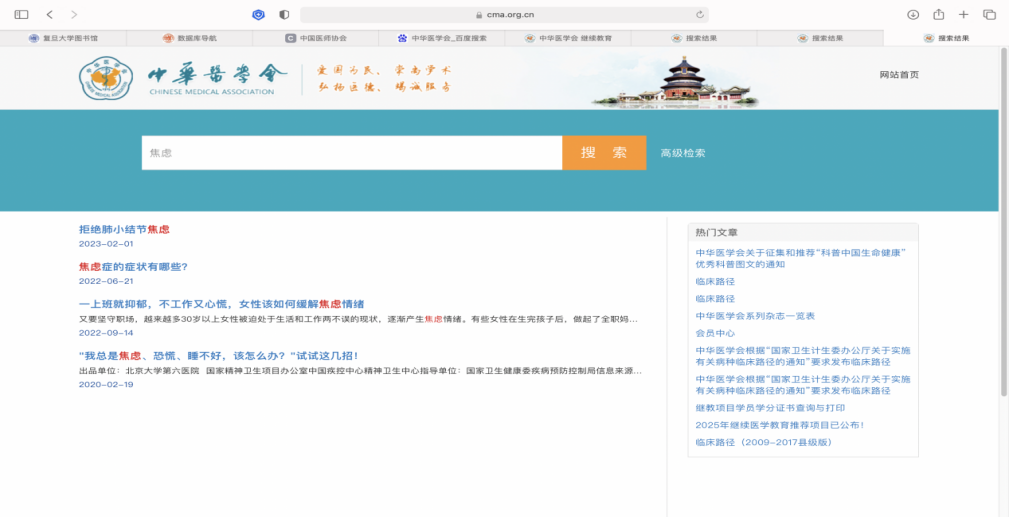


**3.2.9 Chinese Association of Integrative Medicine**

**Search Strategy:** anxiety disorder (1)

**
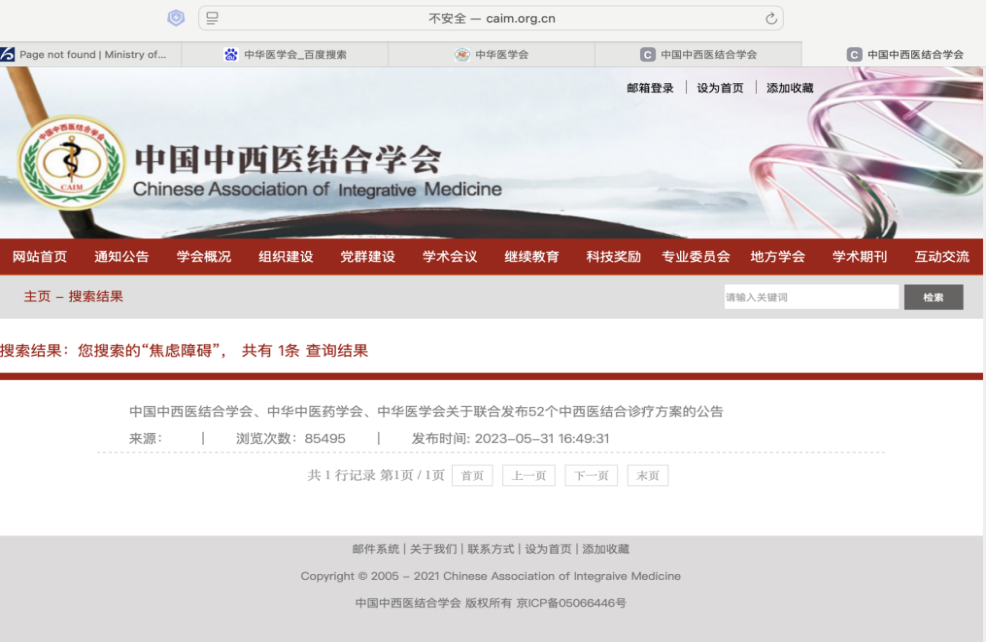
**

**3.3 Databases**

**3.3.1 PubMed**

**Search Strategy:**

1 ((anxiety disorder[Title]) OR (anxiety disorder[MeSH Terms])) OR (anxiety[Title]) (151479)

2 ((guideline*[Title]) OR (consensus*[Title])) OR (recommendation*[Title]) (193269)

3 1 AND 2 (548)


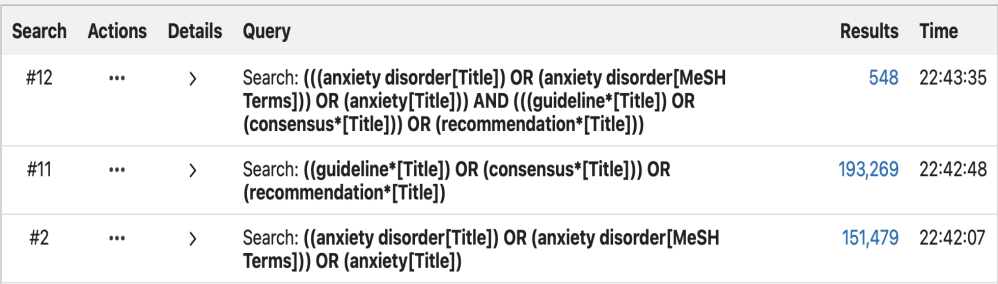

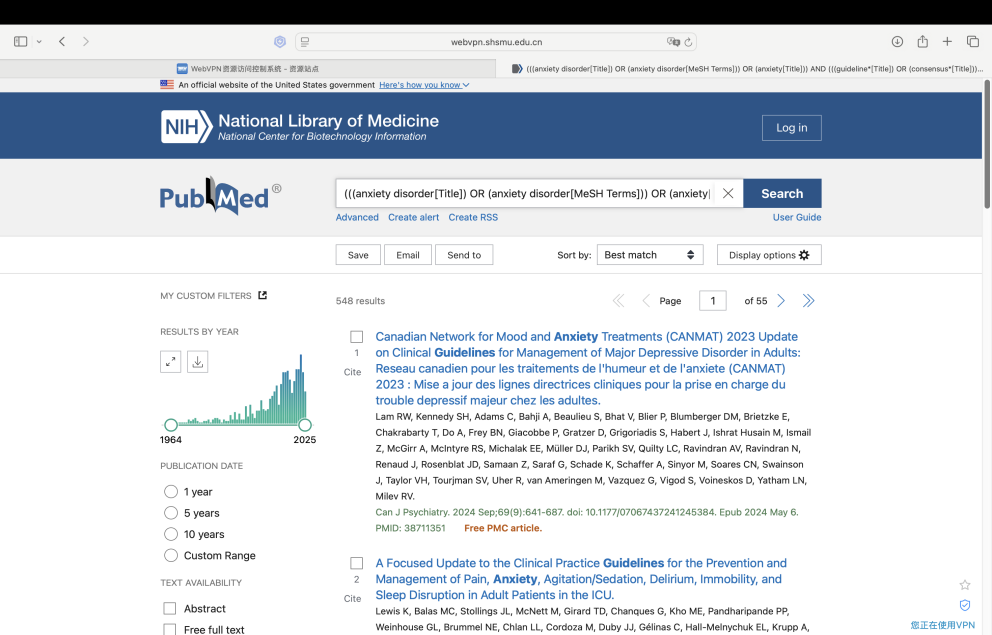


**3.3.2 Web of Science**

**Search Strategy:**

1 (TI=(anxiety disorder)) AND TI=(guideline*) and Preprint Citation Index (Exclude – Database) (136)

2 (TI=(anxiety disorder)) AND TI=(consensus*) and Preprint Citation Index (Exclude – Database) (32)

3 (TI=(anxiety disorder)) AND TI=(recommendation*) and Preprint Citation Index (Exclude – Database) (68)

4 1OR2OR3 (217)


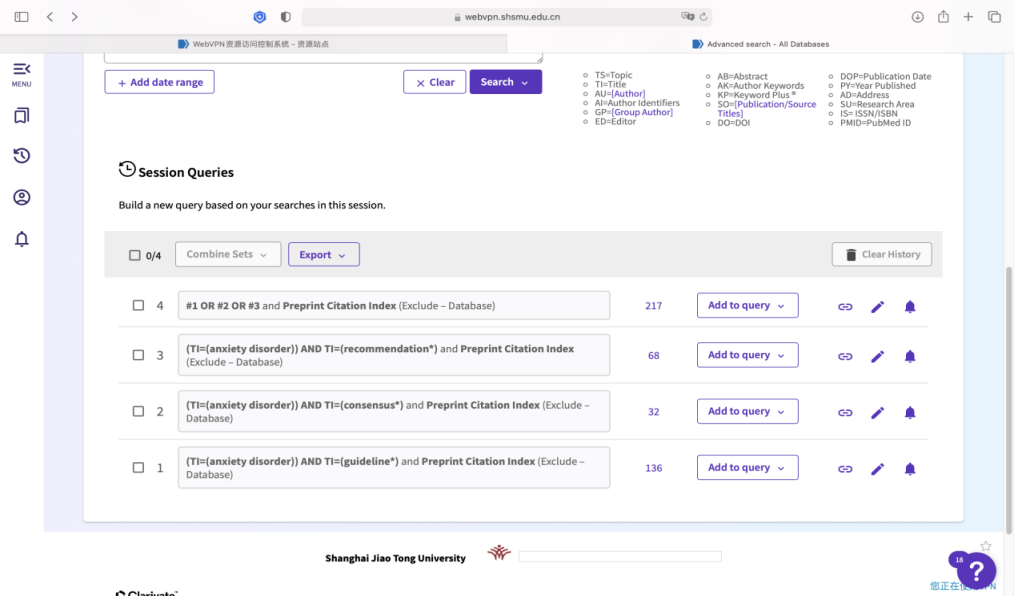


**3.3.3 EMBASE**

**Search Strategy:**

1 (anxiety disorder and guideline*).m_titl. (64)

2 ('anxiety disorder' and consensus*).m_titl. (17)

3 'anxiety disorder'AND recommendation*".m_titl. (0)

4 1 or 2 or 3 (74)


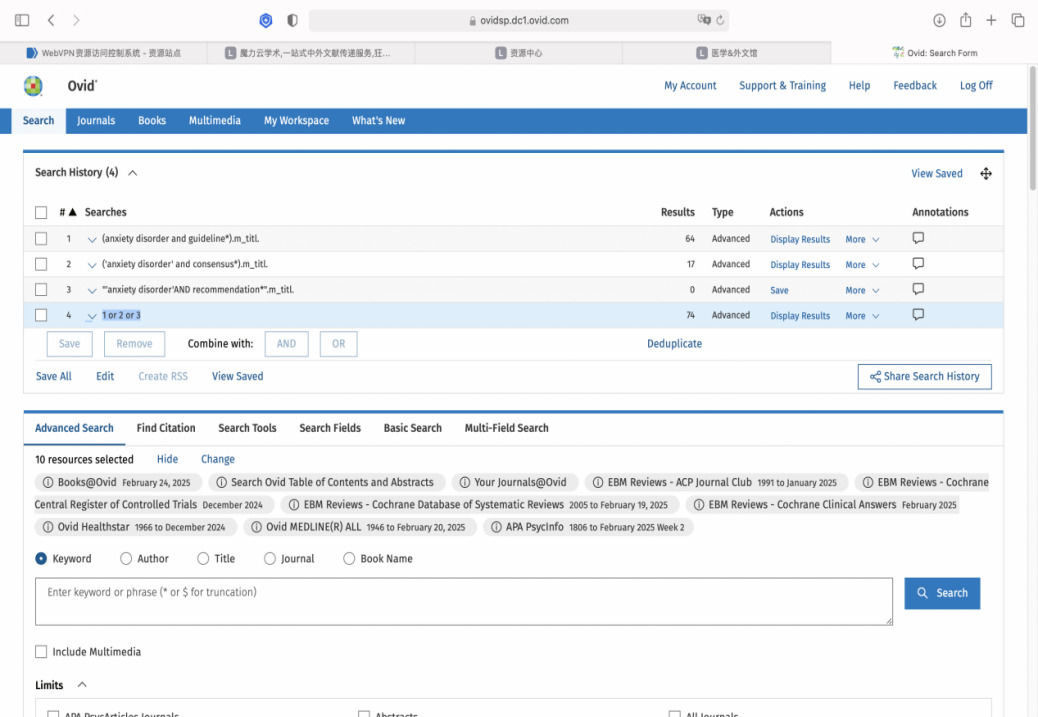


**3.3.4 CINAHL**

**Search Strategy:**


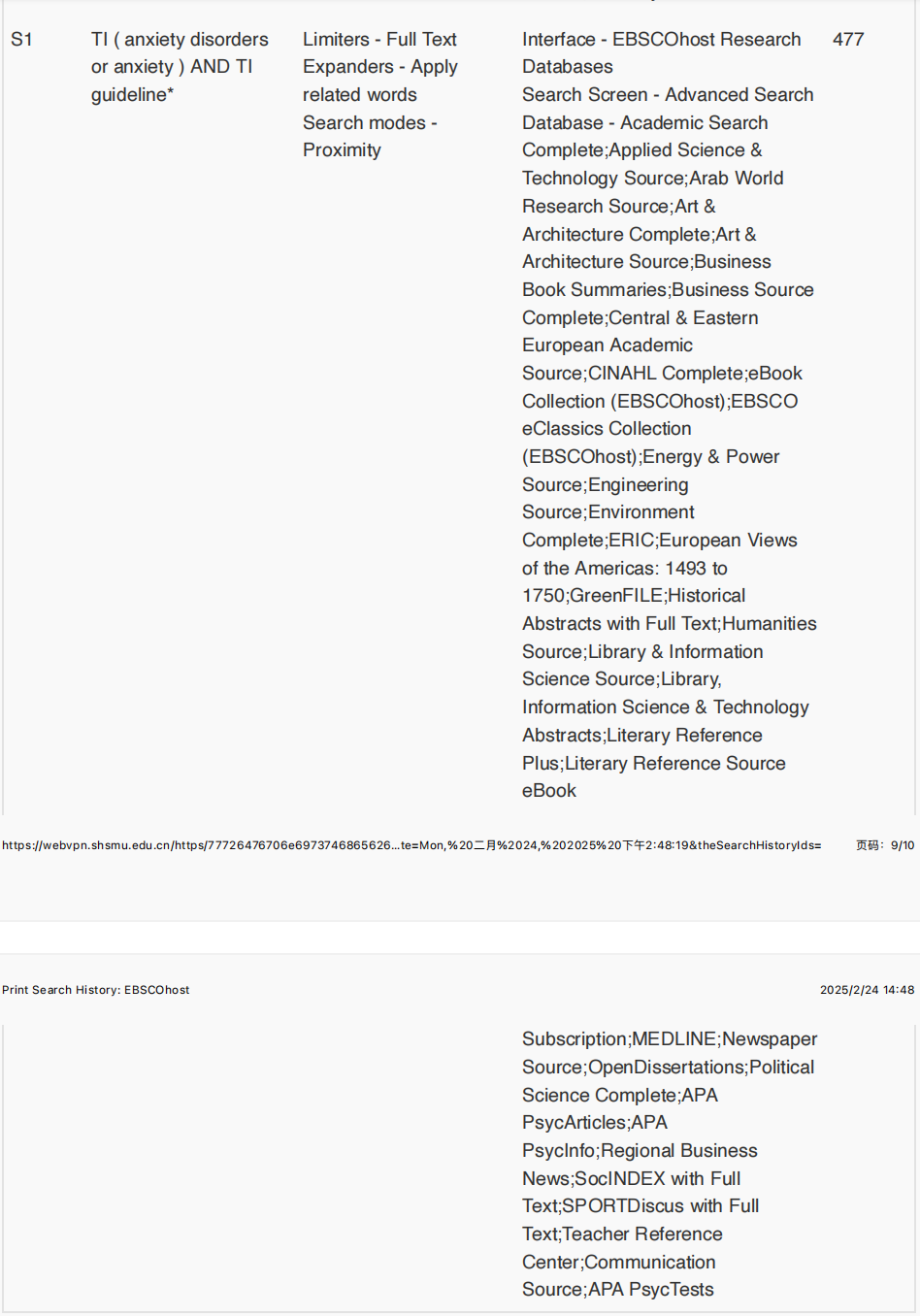


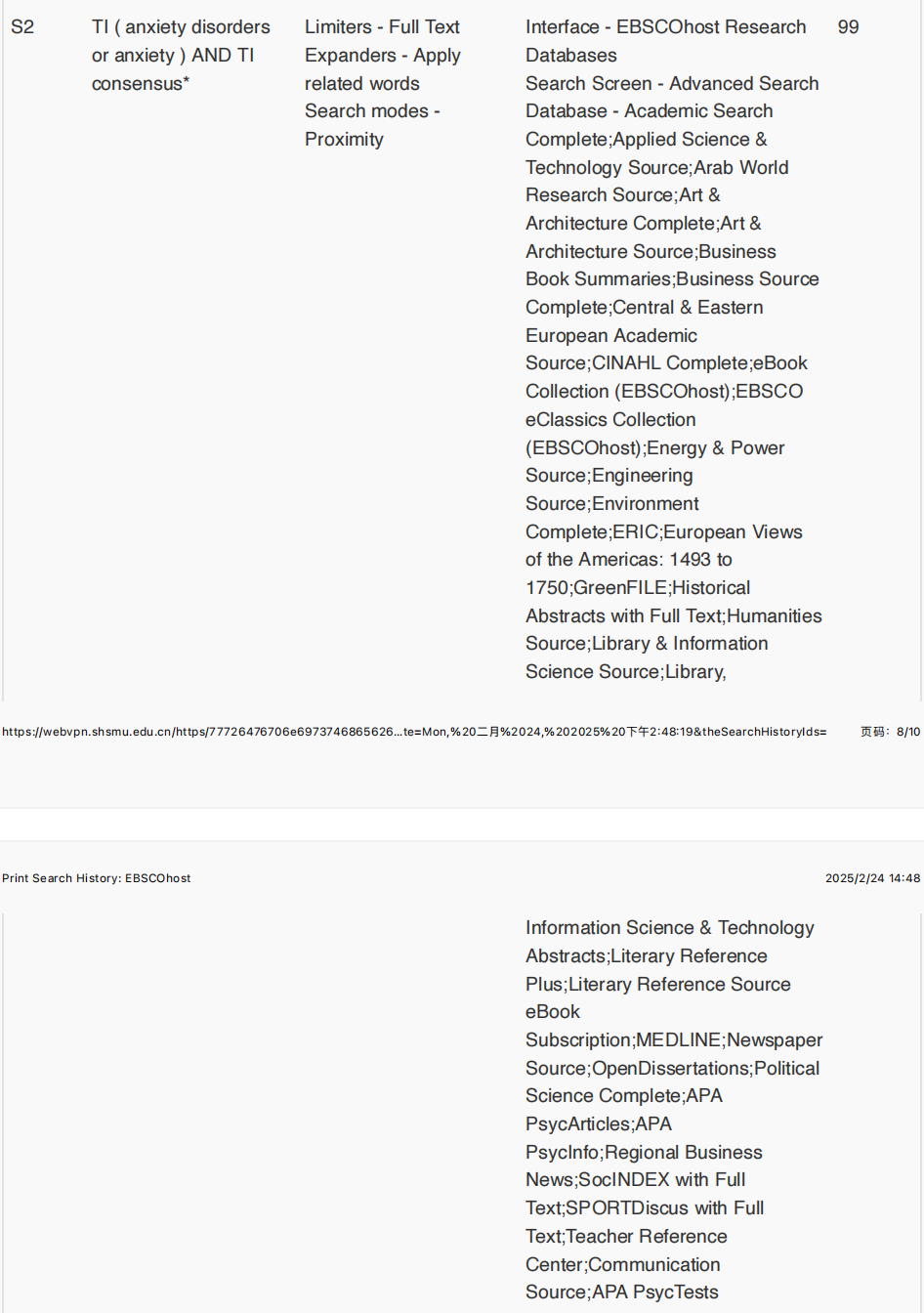


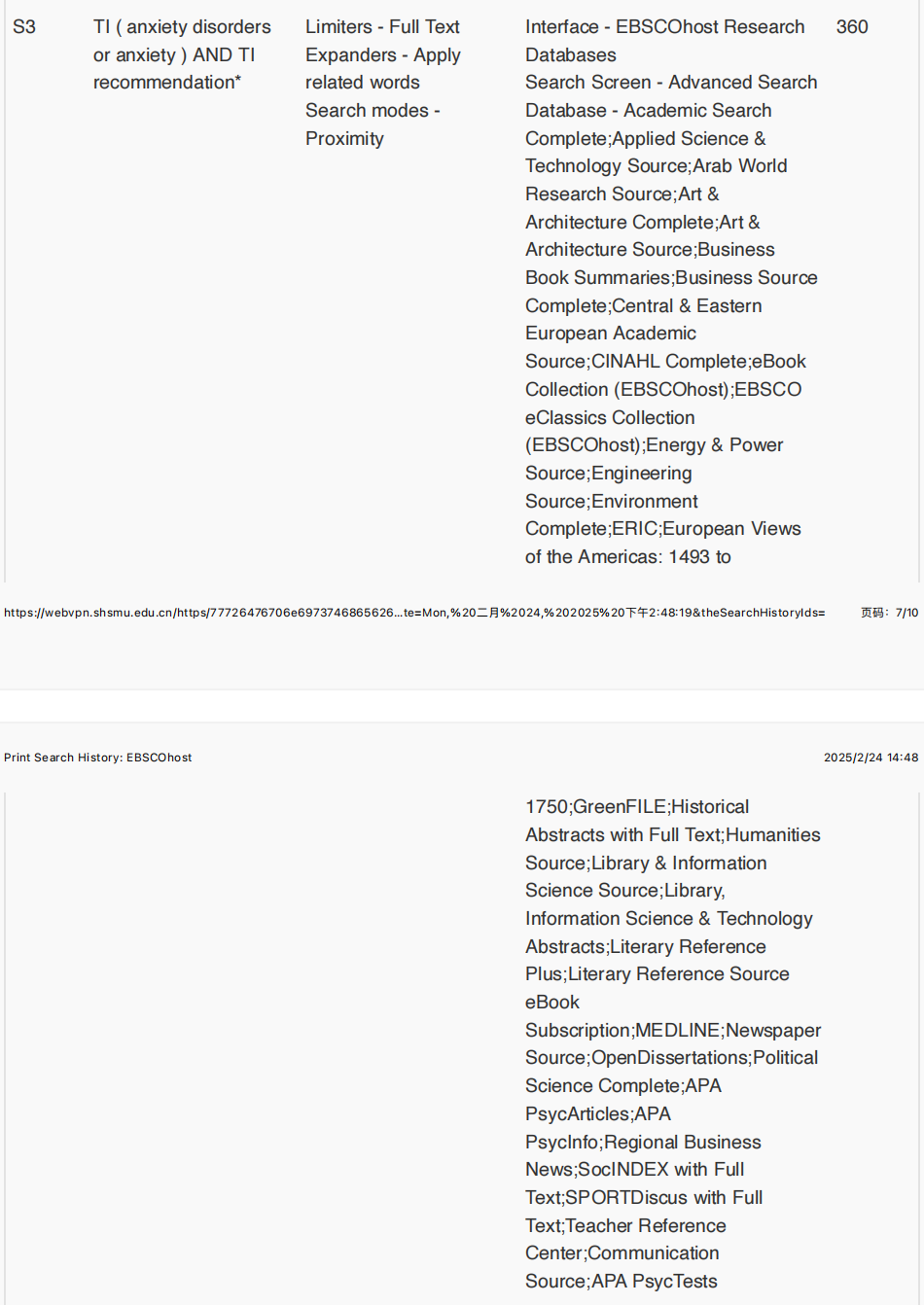


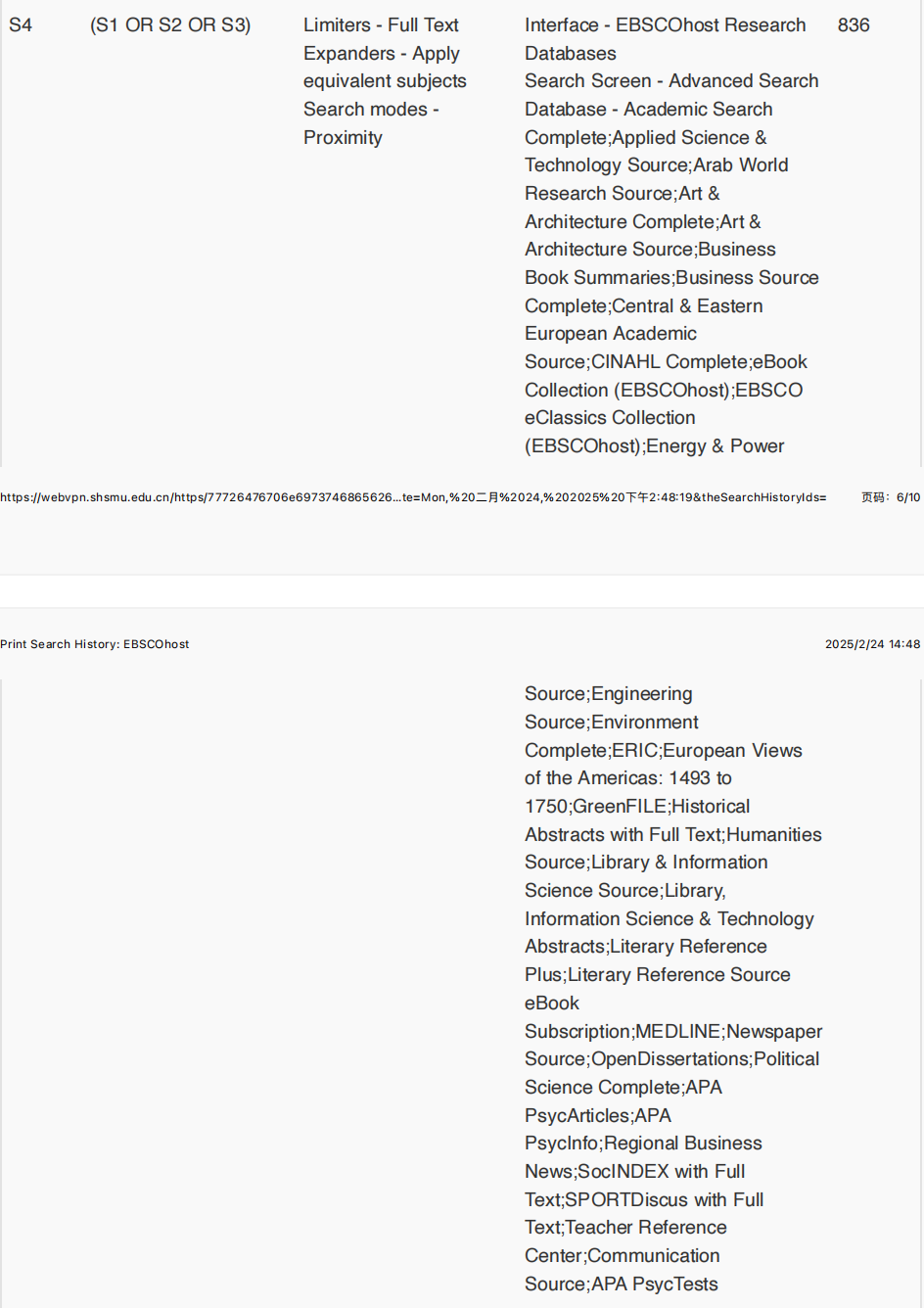


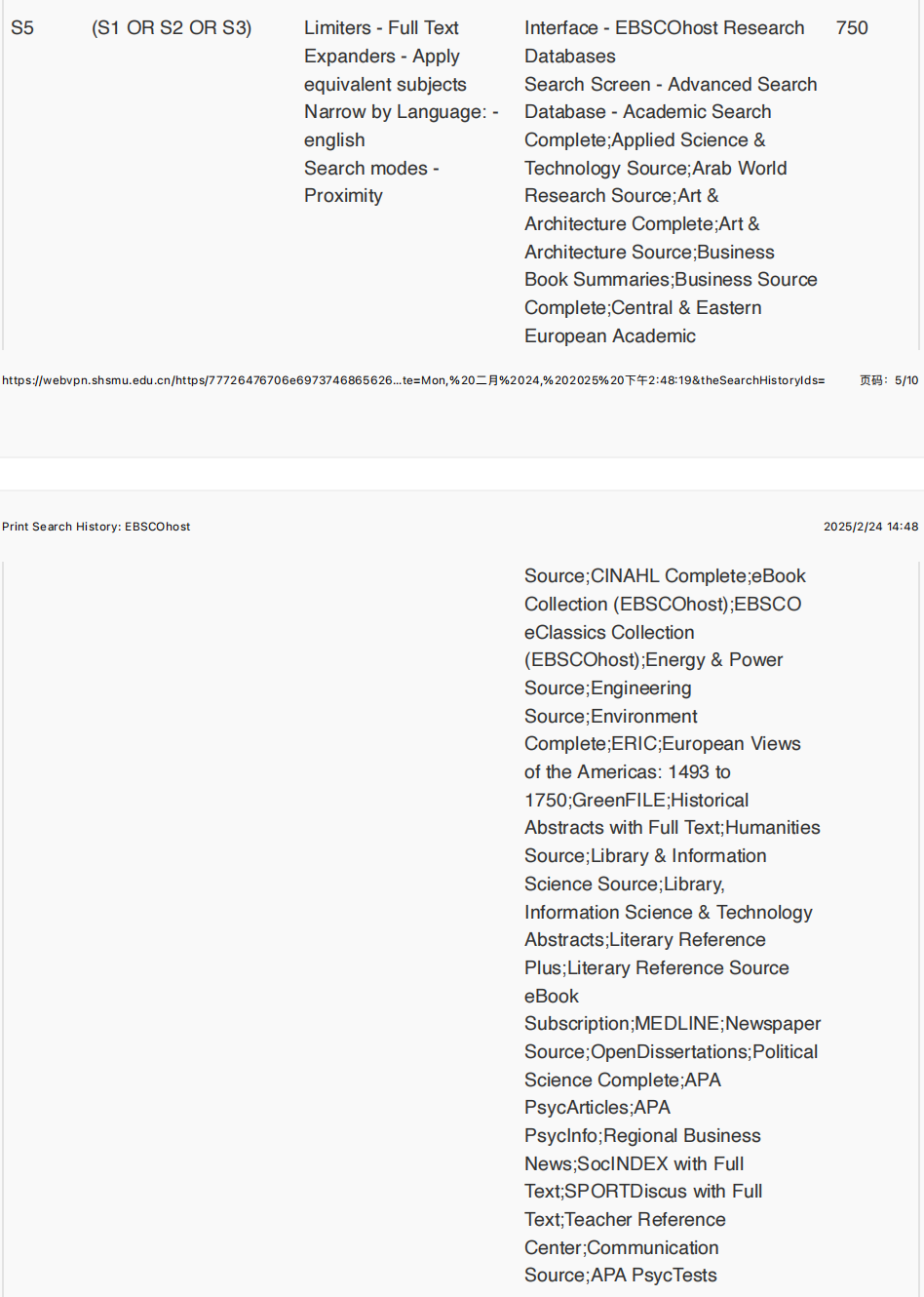


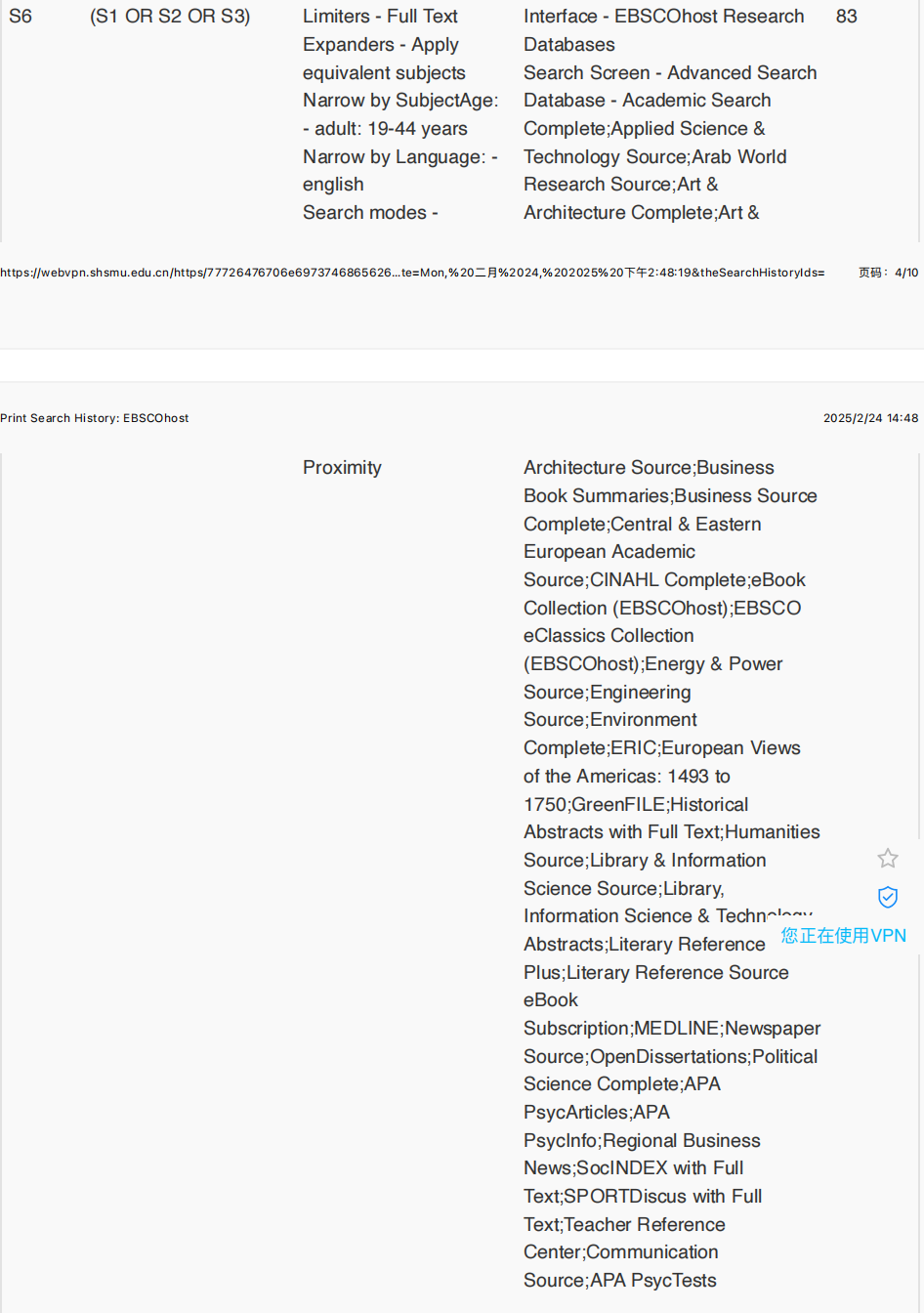


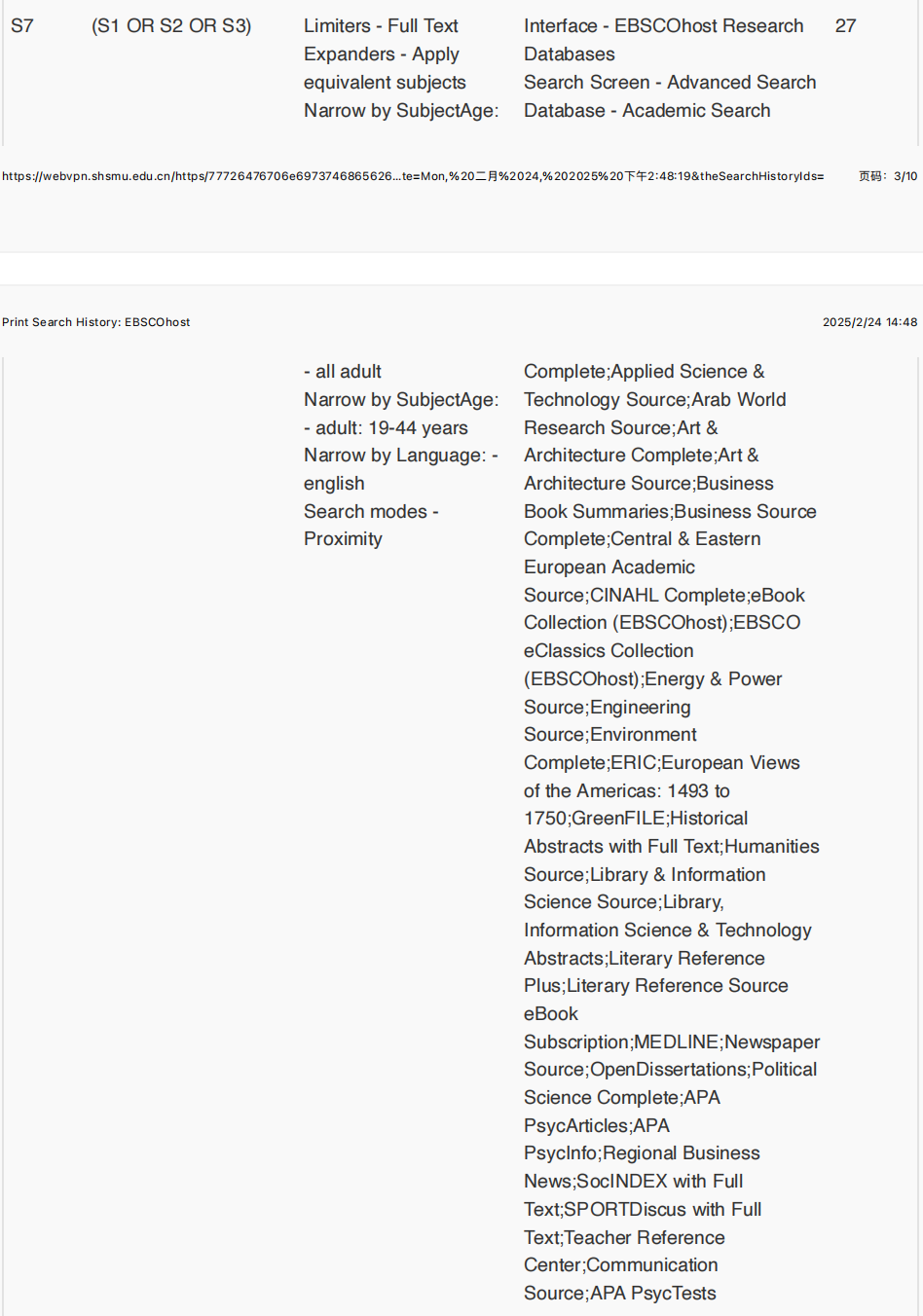


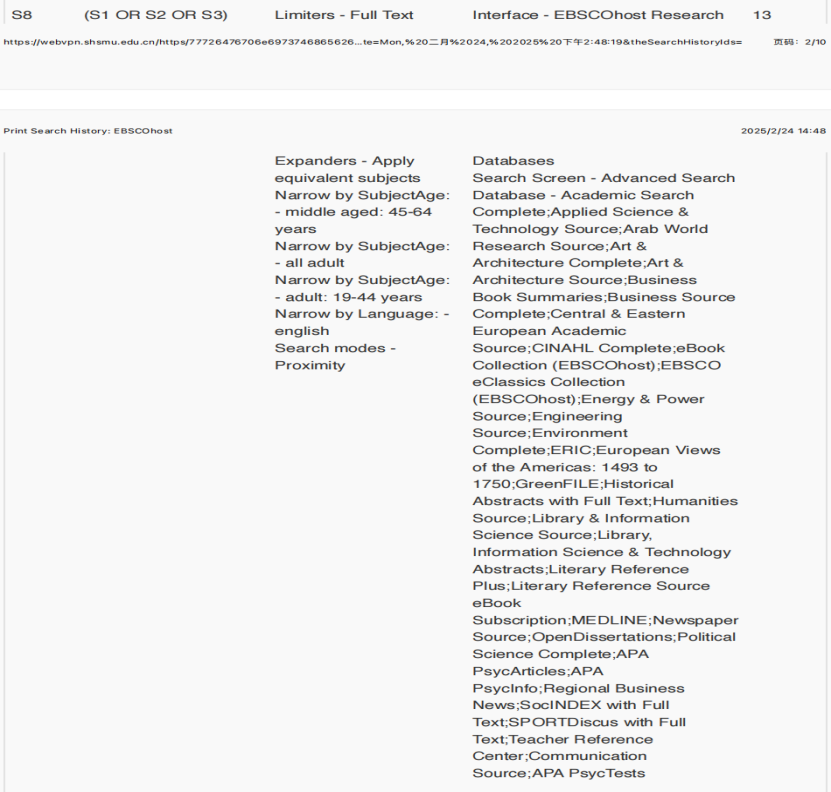


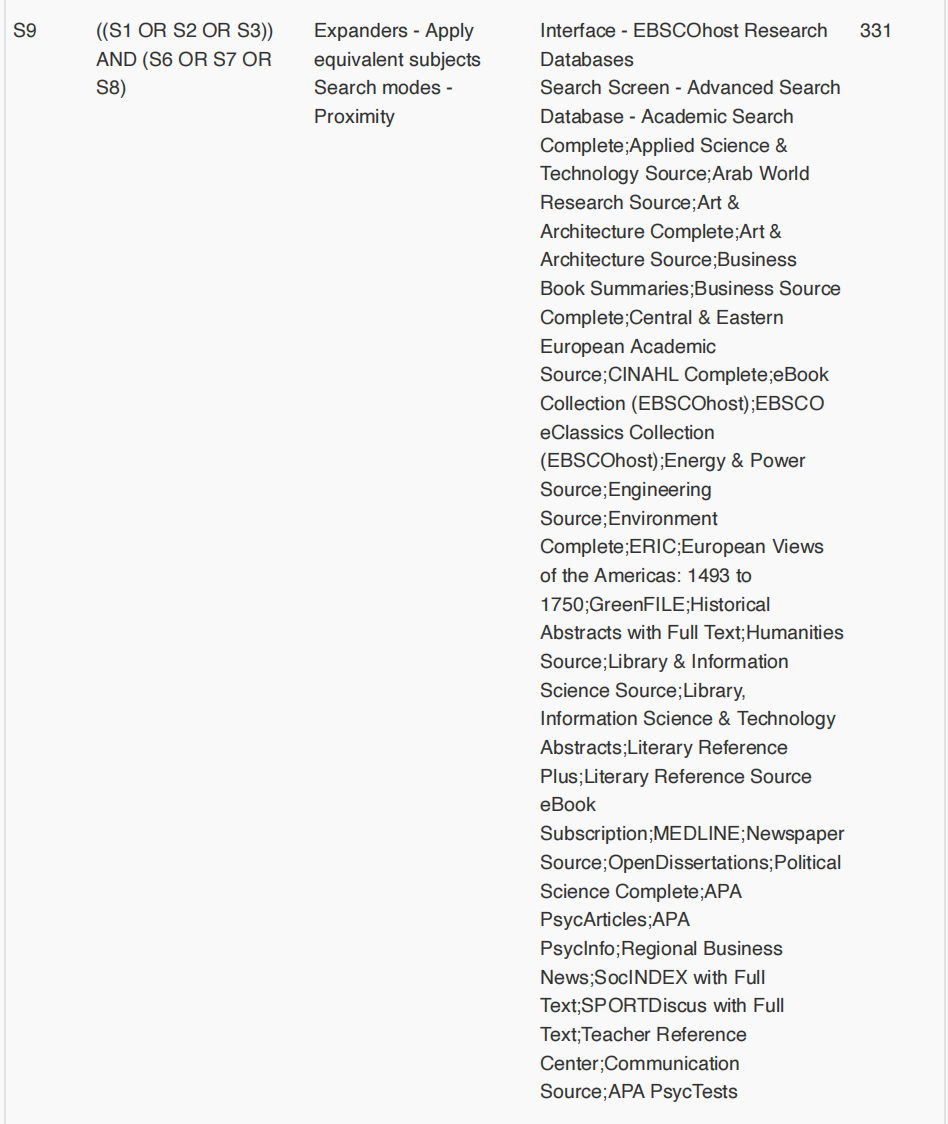


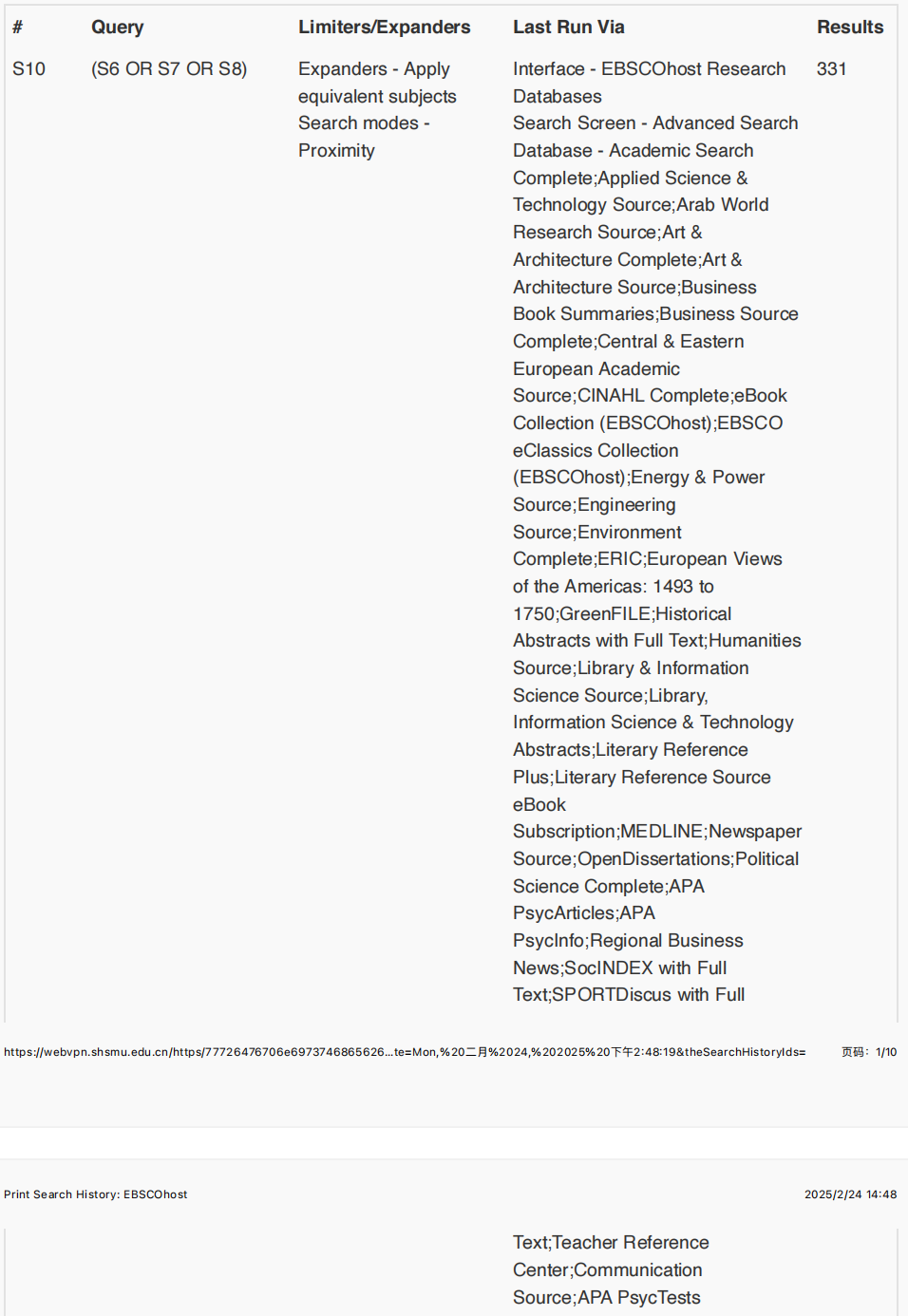


Limiters：Full Text (307)


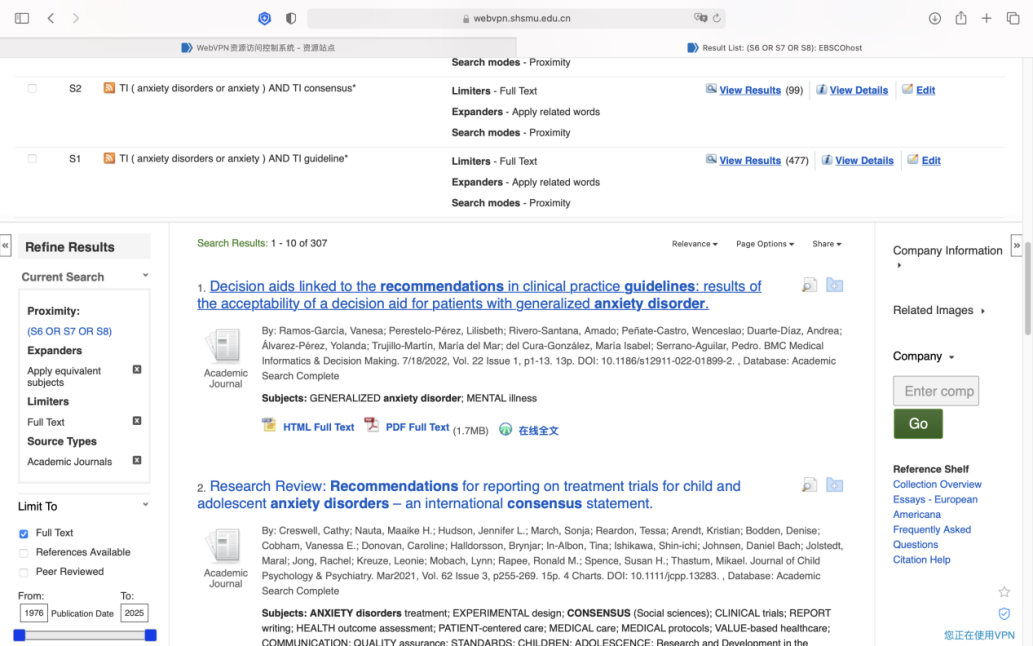


**3.3.5 Cochrane Library**

**Search Strategy:** anxiety disorder in the title, abstract, and keywords (67)


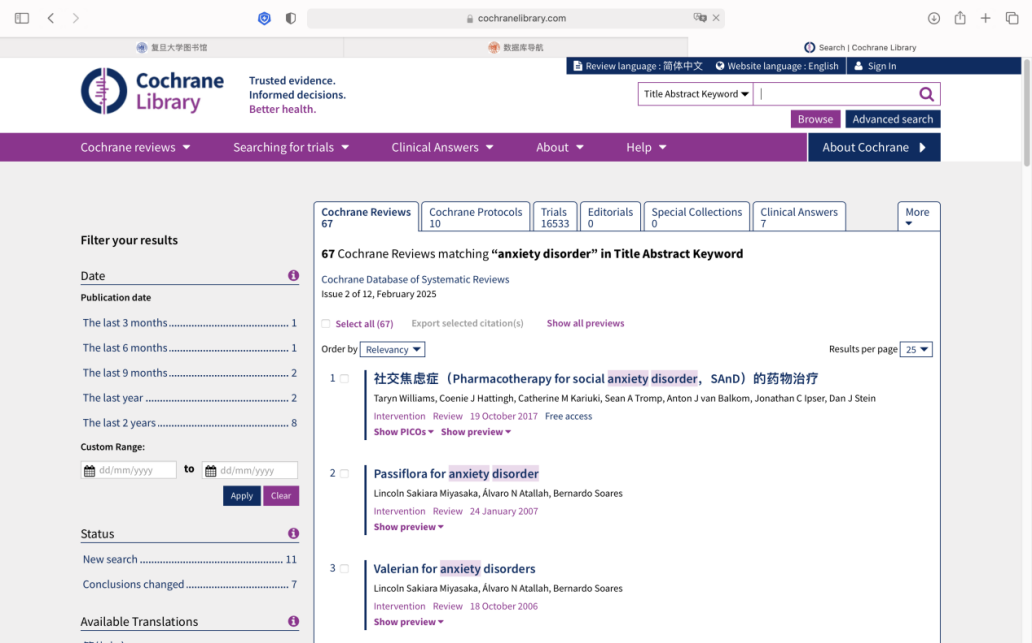


**3.3.6 Joanna Briggs Institute Database**

**Search Strategy:** anxiety disorder (21)


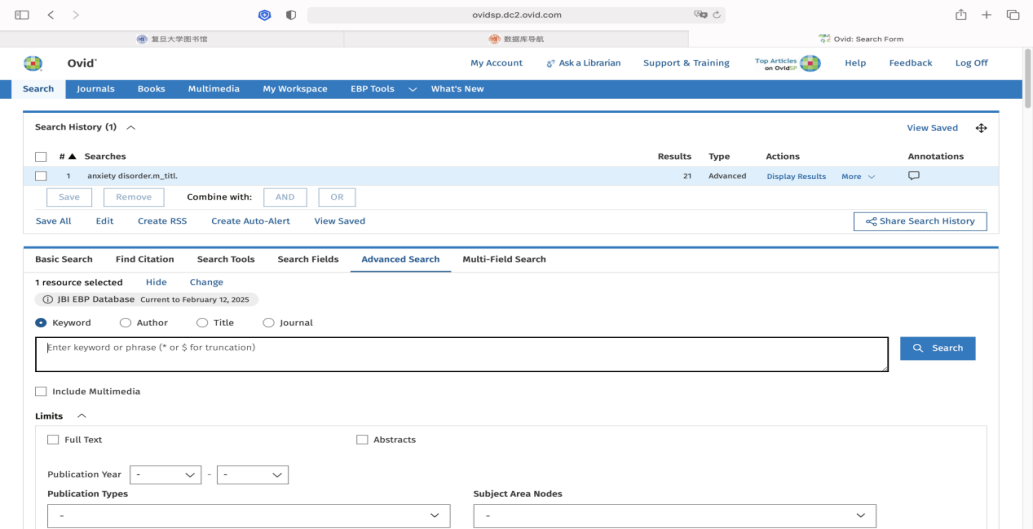


**3.3.7 Wanfang Data Knowledge Service Platform**

**Search Strategy:**

题名或关键词:（“焦虑” or “焦虑障碍”or “焦虑症”） and 题名或关键词:(“指南” or “共识” or “建议” or “推荐意见”） not题名或关键词:(“简介” or “解读”or “释义” or “翻译”or “解读”) (287)


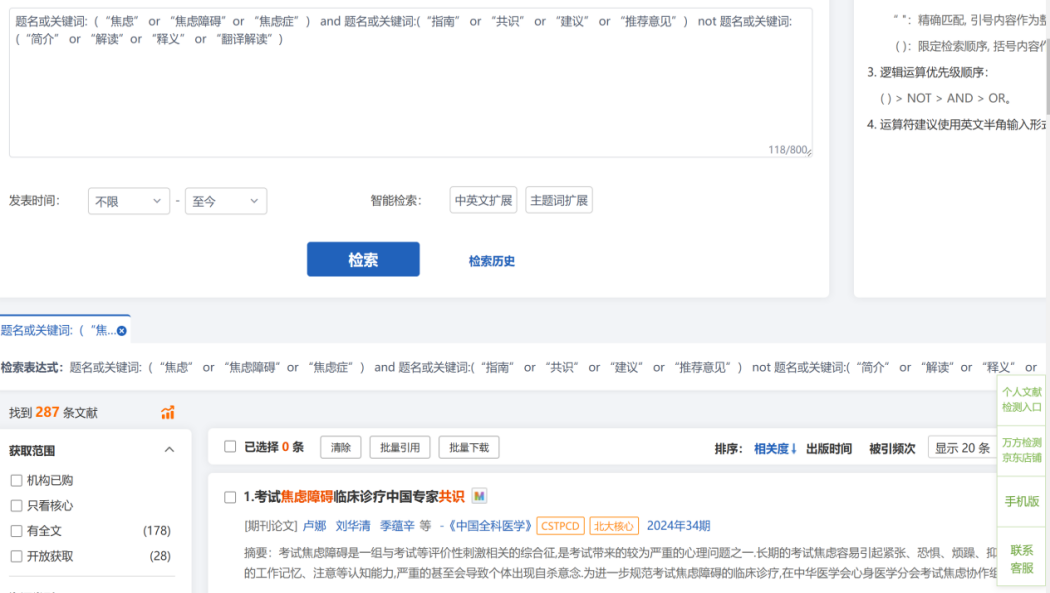


**3.3.8 China National Knowledge Infrastructure**

**Search Strategy:**

SU=（“焦虑”+“焦虑障碍”+“焦虑症”） AND SU=（“指南”+“共识”+“建议”+“推荐意见”） NOT SU=（“解读”+“简介”+“翻译”+“释义”）(71)


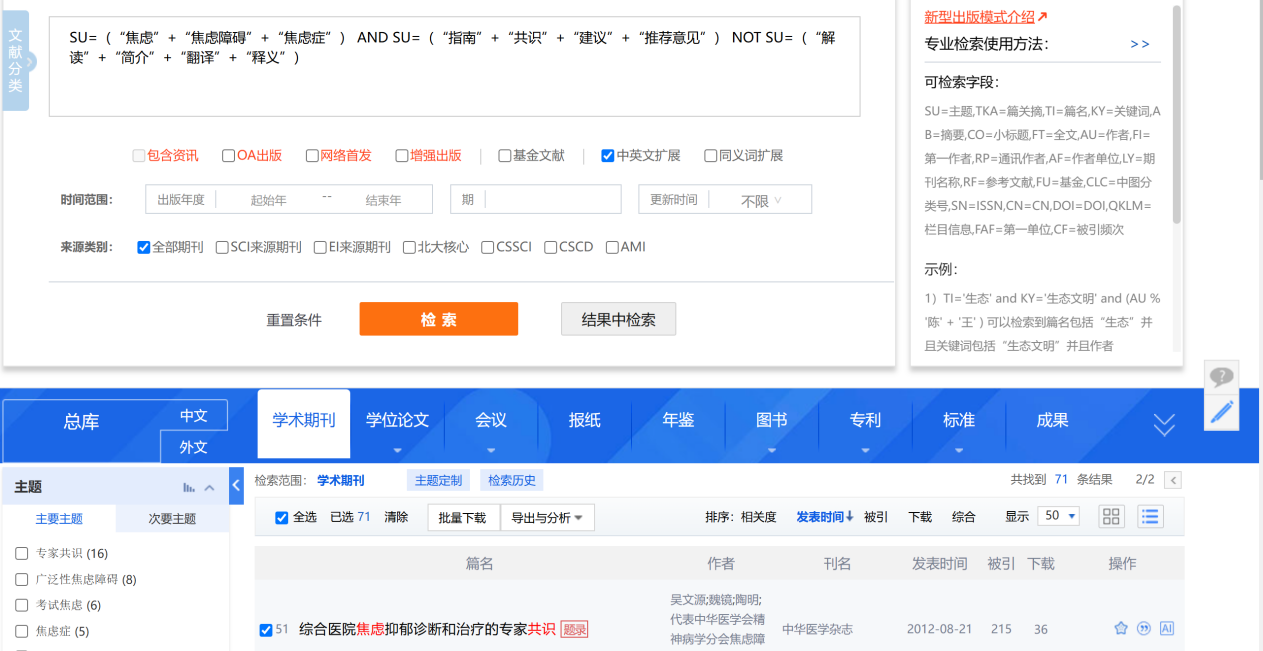


**3.3.9 Chinese Biomedical Literature Service System**

**Search Strategy:**

“焦虑/焦虑障碍/焦虑症”“指南/共识/建议/推荐意见” (87)


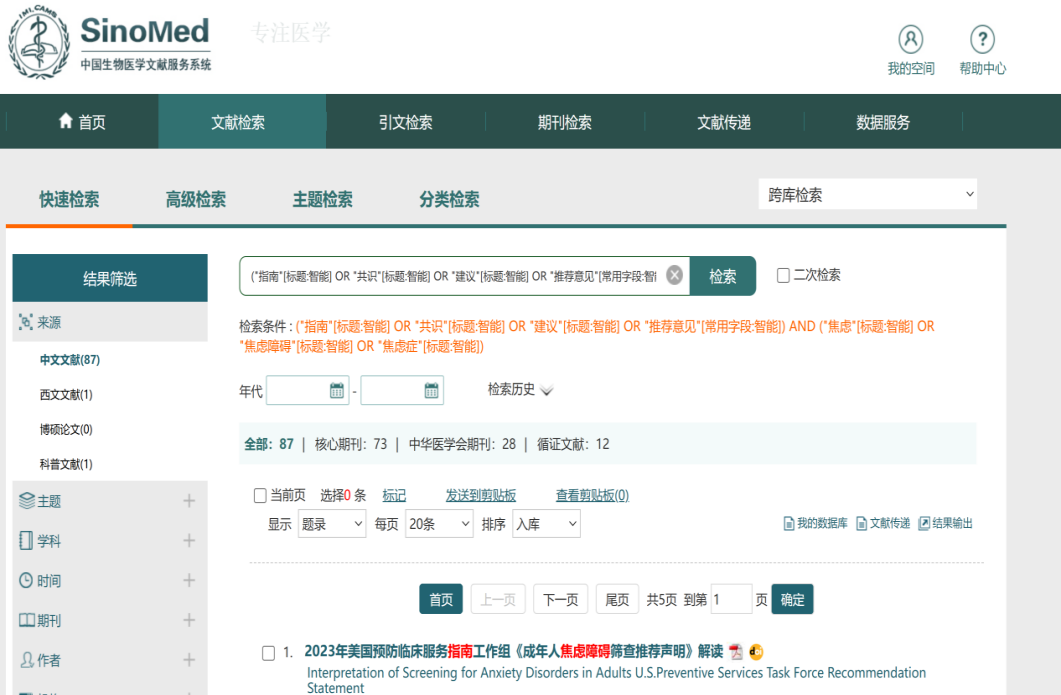


**3.3.10 Chongqing VIP database**

**Search Strategy:**

T=(焦虑 OR 焦虑障碍 OR 焦虑症) AND T=(指南 OR 共识 OR 建议 OR 推荐意见)NOT T=(简介 OR 解读 OR 释义 OR 翻译) (75)


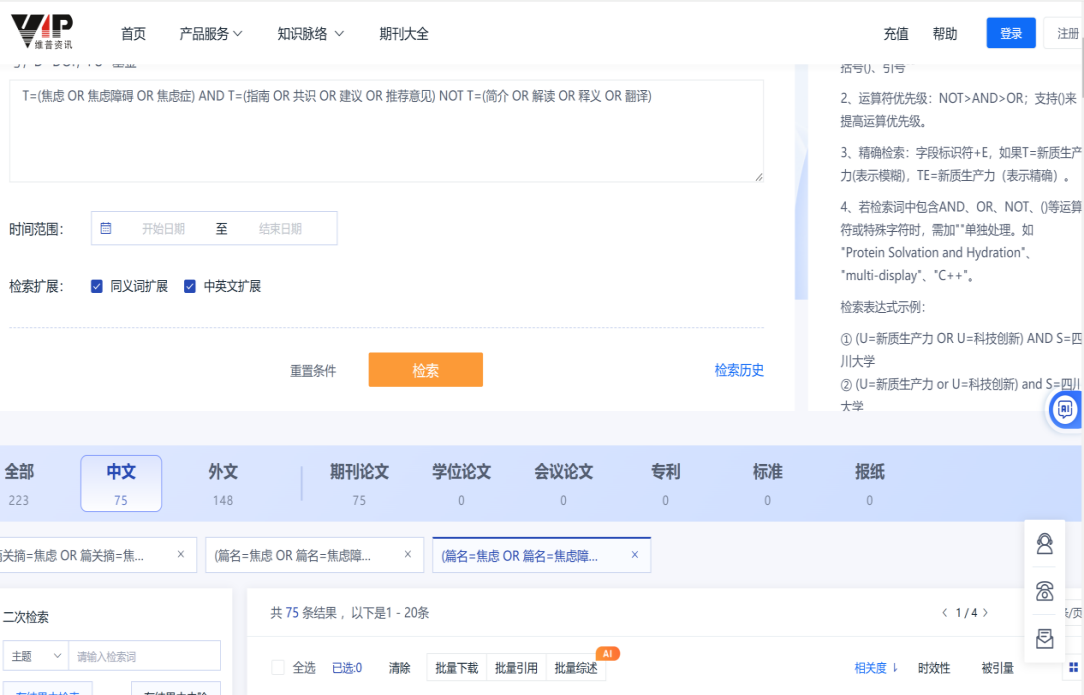

Supplement: Supplementary file 1 [file Supplementaryfile1.docx]
